# Molecular Identification of *Nocardia seriolae* and Comparative Analysis of Spleen Transcriptomes of Hybrid Snakehead (*Channa maculata* Female × *Channa argus* Male) With Nocardiosis Disease

**DOI:** 10.3389/fimmu.2022.778915

**Published:** 2022-01-27

**Authors:** Ning Zhang, Hairui Zhang, Zhongdian Dong, Wei Wang

**Affiliations:** ^1^ Fisheries College, Guangdong Ocean University, Zhanjiang, China; ^2^ Zhongshan Ronghai Aquaculture Co. Ltd., Zhongshan, China; ^3^ Guangxi Key Laboratory of Beibu Gulf Marine Biodiversity Conservation, Beibu Gulf University, Qinzhou, China; ^4^ Guangdong Provincial Key Lab of Pathogenic Biology and Epidemiology for Aquatic Economic Animals, Guangdong Ocean University, Zhanjiang, China

**Keywords:** hybrid snakehead, *Nocardia seriolae*, RNA-seq, fish, immune response

## Abstract

Hybrid snakehead (*Channa maculata* female × *Channa argus* male) is a new freshwater aquaculture fish species in southern China. During intensive aquaculture, hybrid snakeheads are often infected by *Nocardia seriolae*. In this study, hybrid snakehead infected suspiciously by *N. seriolae* in an artificial breeding pond were examined. Diseased hybrid snakeheads swam slowly without food intake, and the clinical symptoms included skin wound, anal swelling and ascites, and white granulomatous in liver, spleen, and kidney of fish. Through bacterial isolation, 16S rDNA sequencing, fluorescence *in situ* hybridization (FISH) and artificial infection experiment, the pathogen was identified as *N. seriolae*. Furthermore, the spleen samples from diseased and healthy male hybrid snakeheads in the same pond were used for RNA-Seq analysis. A total of 3,512 unique transcripts (unigenes) were identified as differentially expressed genes (DEGs), and 1,886 of them were up-regulated in diseased fish. The expression patterns of 20 DEGs were verified by quantitative polymerase chain reaction (qPCR). Several immune-related pathways and many immune-related genes were identified. qPCR results showed that the expression patterns of immune-related genes in the liver and kidney of diseased fish were comparable to that in the spleen. This study provides deep-sequencing data of hybrid snakehead spleen and will help understand the immune response of hybrid snakehead to *N. seriolae*. It is also helpful for the biomarker screening of fish-borne *Nocardia* spp. and the breeding of *nocardiosis*-resistant fish species.

## Introduction

Hybrid snakehead (*Channa maculata* female × *Channa argus* male) is the first generation of hybrid female *C. maculata* and male *C. argus*, which is characterized by fast growth, low oxygen tolerance, high yield, tender meat, and delicious taste. It has become a popular farmed fish in southern China (1–4). In the breeding process, hybrid snakehead often suffers from *Nocardio seriolae* infection, which leads to the outbreak of nocardiosis disease and reduces the yield (5).

*Nocardia* spp. are filamentous and Gram-positive bacteria found in nature as soil saprophytes (6). They are opportunistic pathogens, and some species are pathogenic to fish (7–9). Fish nocardiosis is a systemic disease with the main clinical symptoms of skin ulceration and granuloma formation of internal organs (6). *N. asteroids* (in neon tetras, *Hyphessobrycon innesi*, 1963) (10), *N. seriolae* (in yellowtails, *Seriola quinqueruiata* and *S. purpurascens*, 1968) (11) and *N. salmonicida* (in blueblack salmon, *Oncorhynchus nerca*, 1999) (12) are harmful to fish. Of these, *N. seriolae* is the most common pathogen, which has been found in yellowtail (*S. quinqueradiata*) (13), largemouth bass (*Micropterus salmoides*) (14, 15), striped bass (*Micropterus salmoides*) (16), snakehead (*Ophiocephalus argus Cantor*) (17), grey mullet (*Mugil cephalus*) (16), Japanese flounder (*Paralichthys olivaceus*) (18), hybrid snakehead (5), Japanese sea bass (*Lateolabrax japonicus*) (19, 20) and Japanese eel (*Anguilla japonica*) (8) since its first discovery in 1968. *Nocardia* has a long onset period, high infection rate and mortality rate, which is harmful to the aquaculture industry (6, 17).

Fish mainly rely on innate or adaptive immunity to resist pathogens (21). The infection routes of *N. seriolae* mainly include gills, muscles, and injury sites (6, 22). In naturally infected yellowtails, white spots of nocardiosis were observed in gills, muscles, and skins. In artificial infection experiment, yellowtails infected with *N. seriolae* by intraperitoneal injection (40-100%) and immersion (20-100%) had higher mortality rates than those infected by dermal injection (<60%), while the fish infected *via* oral administration had the lowest mortality rate (<35%) (22). However, there are no effective prevention and control measures for *N. seriolae* infection, and its pathogenic mechanism is still unclear.

RNA-Seq technology has the advantage of obtaining all gene expression patterns of a sample quickly and economically (23). It has been used to screen immune-related genes after infection of various pathogens in fish, including largemouth bass (24), orange-spotted grouper (*Epinephelus coioides*) (25) and hybrid snakehead (5). In previous studies, artificial infections were carried out in fish by injection under laboratory conditions, which may not be consistent with the route of pathogenic infection in aquaculture environment. In order to reflect the innate and adaptive immune mechanisms of fish against pathogenic infections in aquaculture, it is necessary to analyze pathogenic infections that occur in naturally diseased fish in the pond.

In this study, some hybrid snakeheads suspiciously infected with *N. seriolae* were found in a breeding pond in Zhongshan City, Guangdong Province, China. This study aimed to identify and characterize the disease-causing pathogen and gain more insight into the molecular mechanisms of fish immune response to the disease. We conducted histological analysis in diseased fish and artificial infection experiment using healthy hybrid snakeheads. In addition, we performed RNA-Seq analysis on spleen of both healthy and diseased fish, verified the accuracy of RNA-seq by quantitative polymerase chain reaction (qPCR) and examined the expression patterns of some immune genes in liver and kidney.

## Methods and Materials

### Fish Sample Collection and Isolation of Pathogenic Bacteria

All experimental protocols in this study were approved by the Animal Research and Ethics Committee of Guangdong Ocean University Zhanjiang, Guangdong, China (201903003). The study does not involve endangered or protected species. Date of approval: 2 May 2019.

Hybrid snakeheads were collected from a fish farm in Zhongshan City, Guangdong Province, China. During the breeding process, some hybrid snakeheads swam slowly to the side of the pond without food intake. Most of these had wounds on their body surface. Abdominal swelling was observed, and death occurred. It was suspected that these fish were suffering from nocardiosis. Five diseased male hybrid snakeheads (body weight of 1250 ± 318 g) and five healthy male hybrid snakeheads (body weight of 1176 ± 227 g) in the same pond were anesthetized with 100 mg/L tricaine methanesulfonate (MS222) (Sigma, USA). Tissue parts of the spleen, liver, and kidney of two individuals were inoculated in brain-heart immersion medium (BHI) (Huankai, Guangzhou, China) in a sterile environment and placed in an incubator at 28°C for inverted culture. Other tissues of the spleen, liver and kidney were dissected from the other three fish and divided into two groups. One group of the tissues was quickly frozen in liquid nitrogen and stored at -80°C for RNA and DNA extraction, and the other group of the tissues was placed in 4% paraformaldehyde (PFA) for histological analysis and fluorescence *in situ* hybridization (FISH).

### Histological Analysis of Hybrid Snakehead and Identification of Pathogenic Bacteria

Spleen, liver, and kidney samples from healthy and diseased fish groups (n=3) were collected and fixed in 4% PFA for 24 h and dehydrated using a series of ethanol (Sangon Biotech, Shanghai, China). The samples were then cleared by xylene (Sangon Biotech, Shanghai, China) and embedded using paraffin (Sangon Biotech, Shanghai, China). Sections were cut at 8 µm thickness and stained with hematoxylin and eosin (H&E) (Sangon Biotech, Shanghai, China). After 7 d of bacterial culture, a single colony was selected and inoculated into a BHI liquid medium, placed in a shaking incubator, and incubated at 28°C (170r/min) for 4 d. A small amount of bacterial liquid was taken for PCR identification using 16s-F and 16s-R (**Supplementary Table 1**) primers (26). PCR products were sent to Genewiz Company (Suzhou, China) for sequencing. The sequences were analyzed by DNASTAR 7.10 software and blastn tool (https://blast.ncbi.nlm.nih.gov/Blast.cgi). The remaining bacterial solution was centrifuged to discard the supernatant, and the bacteria were resuspended with sterile phosphate-buffered solution (PBS). Twenty healthy male fish were randomly selected from the aquaculture pond, and ten individuals were injected with 200 uL bacterial suspension intraperitoneally. Other ten individuals were injected with 200 μL PBS as the control group and then placed in 200 L water tanks for observation of clinical symptoms. The fish were fed with a compound pelleted diet (Tongwei, Chengdu, China) once a day (1% of biomass).

### Fluorescence *In Situ* Hybridization (FISH)

The 16S rDNA sequencing results were analyzed, and a DNA probe (**Supplementary Table 1**) labeled with Cy3 at the 5’-end was designed for FISH in spleen, liver, and kidney of both diseased and healthy individuals. The paraffin was sliced through the slicer and oven-roasted at 62°C for 2 h. The slices were soaked in xylene (2 changes, 15 min each) and dehydrated using pure ethanol (2 changes, 5 min each). Then, the slices were dehydrated by gradient ethanol of 85% and 75% for 5 min each. The slices were washed using diethyl pyrocarbonate (DEPC) treated water dilution, boiled in the retrieval solution for 10-15 min and cooled at room temperature. The tissues were marked with a liquid blocker pen, according to the characteristics of tissues. Proteinase K working solution (20 μg/mL, Sangon Biotech, Shanghai, China) was added and incubated at 37°C for 20 min. The tissues were washed with pure water and then washed three times using PBS (pH 7.4), 5 min each. Pre-hybridization solution was added to each section and incubated for 1 h at 37°C. The pre-hybridization solution was removed. The probe hybridization solution (1 μM) was added, and the section in a humidity chamber was incubated and hybridized overnight at 37°C. The hybridization solution was removed, and sections were washed in 2×SSC for 10 min at 37°C, 1×SSC two times for 5 min each at 37°C and 0.5×SSC for 10 min at room temperature. The sections were incubated with DAPI (4′, 6-diamidino-2-phenylindole) (Servicebio, Wuhan, China) for 8 min in the dark. The slices were covered with cover glass, and photographs were taken with a fluorescence microscope. The nucleus stained by DAPI was blue under ultraviolet excitation, and the positive signal of bacteria was red fluorescence-labeled.

### Total RNA Extraction, cDNA Library Construction, and Sequencing

Total RNA of spleen from three healthy and three diseased hybrid snakeheads were separately extracted using Trizol reagent (Invitrogen, Carlsbad, USA) following the manufacturer’s protocol. Genomic DNA (gDNA) was removed using DNase I (TaKaRa, Dalian, China). RNA degradation and contamination were monitored using 1% agarose gels (Sangon Biotech, Shanghai, China). RNA integrity was assessed using the RNA Nano 6000 Assay Kit for the Bioanalyzer 2100 system (Agilent Technologies, California, USA), and RNA concentration was measured using Qubit^®^ RNA Assay Kit in Qubit^®^ 2.0 Flurometer (Life Technologies, California, USA). RNA (1 μg) per sample was used as input material for RNA sample preparation. Sequencing libraries were generated using NEBNext^®^Ultra™ RNA Library Prep Kit for Illumina^®^ (NEB, Massachusetts, USA) following manufacturer’s instructions, and index codes were added to attribute sequences to each sample. Briefly, messenger RNA (mRNA) was purified from total RNA using poly-T oligo-attached magnetic beads. Fragmentation was carried out using divalent cations under elevated temperature in NEBNext First Strand Synthesis Reaction Buffer (5X) (NEB, Massachusetts, USA). First-strand complementary DNA (cDNA) was synthesized using a random hexamer primer and M-MuLV Reverse Transcriptase (M-MLV). Second strand cDNA synthesis was performed using DNA Polymerase I and RNase H (Thermo, Massachusetts, USA). Remaining overhangs were converted into blunt ends *via* exonuclease/polymerase activity. After adenylation of 3’-end of DNA fragments, NEBNext Adaptor with hairpin loop structure was ligated to prepare for hybridization. In order to select cDNA fragments (240 bp), the library fragments were purified with AMPure XP system (Beckman Coulter, Beverly, USA). Then, 3 μL USER Enzyme (NEB, Massachusetts, USA) was used with adaptor-ligated cDNA at 37°C for 15 min, followed by 5 min at 95°C. Then, PCR was performed with Phusion High-Fidelity DNA polymerase (NEB, Massachusetts, USA), universal PCR primers and Index (X) primer. Finally, PCR products were purified (AMPure XP system), and library quality was assessed on the Agilent Bioanalyzer 2100 system (Agilent Technologies Inc, California, USA). Clustering of the index-coded samples was performed on a cBot Cluster Generation System using TruSeq PE Cluster Kit v3-cBot-HS (Illumia, California, USA) according to the manufacturer’s instructions. After cluster generation, the library preparations were sequenced on an Illumina Hiseq 2000 platform, and paired-end reads were generated.

### Quality Control, Transcriptome Assembly and Annotation

The sequences were processed with a bioinformatic pipeline tool, BMKCloud (www.biocloud.net) online platform. Raw data (raw reads) of fastq format were processed through in-house perl scripts. Clean data (clean reads) were obtained by removing reads containing adapter, reads containing ploy-N and low-quality reads from raw data. Q20, Q30, GC-content and sequence duplication level of the clean data were calculated. All clean data (clean reads) were submitted to the sequence read archive (SRA) (https://www.ncbi.nlm.nih.gov/sra) (Accession number: SUB9378553), and all the downstream analyses were based on clean data with high quality. Transcriptome assembly was accomplished using Trinity (v2.5.1) with min_kmer_cov set to 2, and all other parameters were set to default (27). To remove sequence redundancy, the assembled contigs were processed with the CD-HIT and TGICL tools, and sequences with significant similarities (>90%) were clustered into a group. The longest transcripts in different groups were retained in the final assembly, and these unique transcripts were called “unigenes”. The unigenes were then annotated based on the following databases: National Center for Biotechnology Information (NCBI) non-redundant protein sequences (NR), protein family Pfam, EuKaryotic Orthologous Groups (KOG)/Clusters of Orthologous Groups (COG) of proteins, Swiss-Prot (A manually annotated and reviewed protein sequence database), Gene Ontology (GO) and Kyoto Encyclopedia of Genes and Genomes (KEGG).

### Differential Gene Expression Analysis and Enrichment Analysis

Differential expression analysis of spleen from healthy and diseased groups was performed using the DESeq R package (1.10.1). Gene expression levels were estimated with the FPKM values (Fragments Per Kilobase of transcript per Million mapped reads). The p-values were adjusted using Benjamini and Hochberg’s approach for controlling the false discovery rate (FDR). Genes with an adjusted p-value < 0.01 were assigned as differentially expressed. The visualization of similarities and differences between healthy and diseased fish samples was accomplished by principal component analysis (PCA), volcano plot and heat map. GO enrichment analysis of the differentially expressed genes (DEGs) was implemented by the topGO R package. Kolmogorov-Smirnov test was done, and KOBAS software (28) was used for the statistical enrichment of DEGs in KEGG pathways.

### qPCR Validation

In order to validate the results of the RNA-Seq, twenty DEGs were randomly selected for validation by qPCR. Primers were designed according to the transcriptome sequences (**Supplementary Table 1**). qPCR was performed on Roche LightCycler 96 (Roche, Forrentrasse, Switzerland) using SYBR^®^ Green qPCR Mix (GDSBio, Guangzhou, China). The reaction was carried out using a qPCR mixture of 15.0 μL, containing 7.5 μL 2 X SYBR^®^ Green qPCR, 0.6 μL of each forward and reverse primer, 1.5 μL cDNA and 4.8 μL ddH_2_O. qPCR amplification was done as follows: 180 s at 95°C for pre-incubation, followed by 40 cycles at 95°C for 15 s, 60°C for 15 s and 72°C for 30 s. Dissociation and melting curves of qPCR products were performed, and results were analyzed. *β-actin* and *ef*2*b* were used as reference genes to determine relative expression (5). The transcriptional data were analyzed by the 2^-ΔΔCt^ method, and the data were edited by GraphPad Prism 8 (expressed as log_2_ transformed values).

## Results

### Clinical Symptoms of Diseased Hybrid Snakehead and Histological Changes

**Figure 1:**
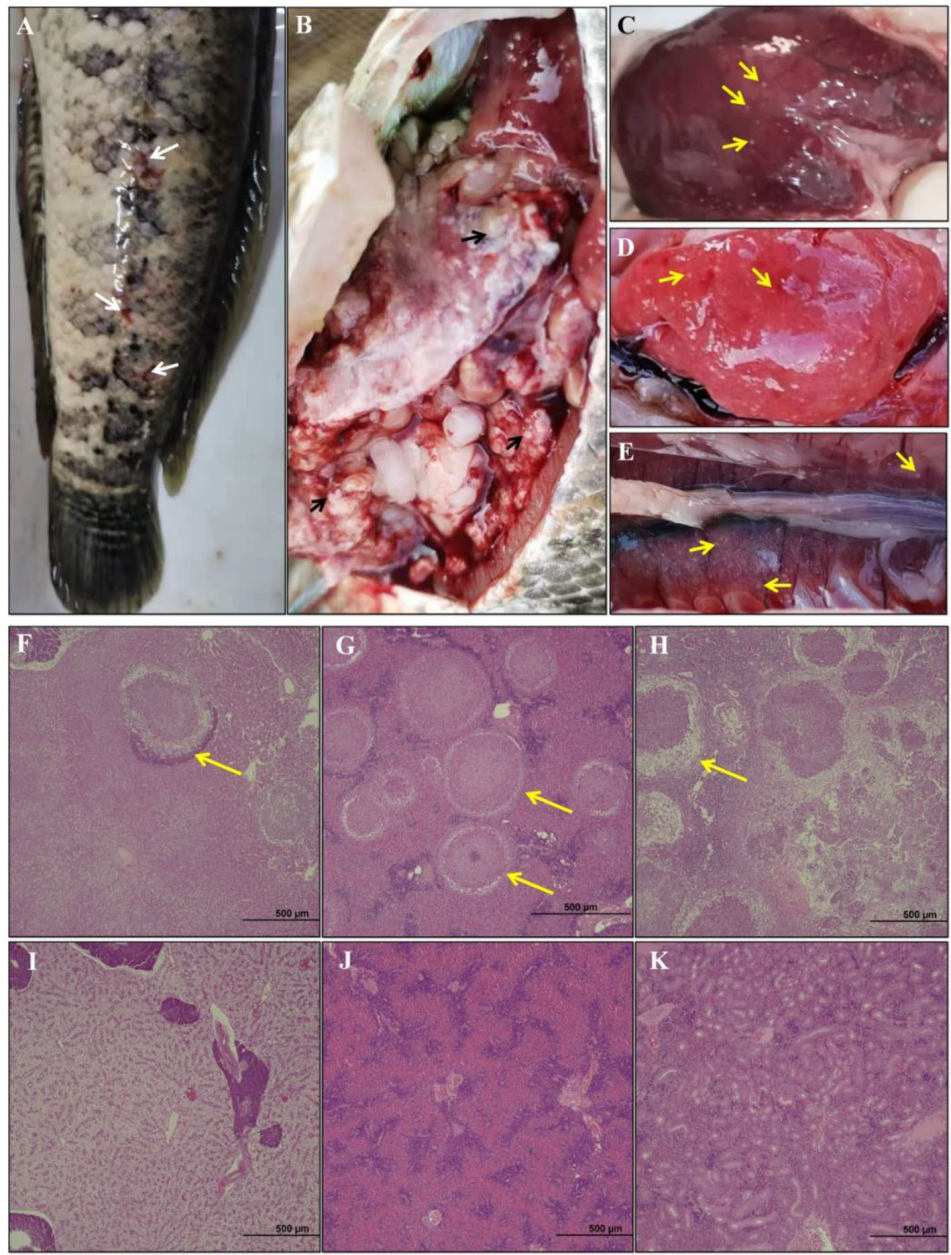
**(A)** Clinical signs of diseased hybrid snakehead body surface. Appearance of abdominal cavity **(B)**, liver **(C)**, spleen **(D)** and kidney **(E)**. Histopathological sections of hybrid snakehead were shown. **(F–H)** correspond to the liver, spleen, and kidneys of the diseased fish; **(I–K)** correspond to the liver, spleen, and kidneys of the healthy fish. The white arrows indicate superficial lesions, black arrows indicate granules of *N. seriolae*, and yellow arrows indicate the necrosis and granuloma.

In this study, skin wound, anal swelling and ascites were observed in diseased hybrid snakehead (**Figure 1A**). Some seriously affected fish were found dead. Many white granulomatous cysts were observed on the epidermis of the liver (**Figure 1C**), spleen (**Figure 1D**) and kidney (**Figure 1E**) of diseased fish. Compared with the healthy hybrid snakehead, granulomatous inflammation in the liver (**Figure 1F**), spleen (**Figure 1G**) and kidney (**Figure 1H**) of diseased hybrid snakehead was observed.

### Isolation and Identification of Pathogenic Bacteria

**Figure 2:**
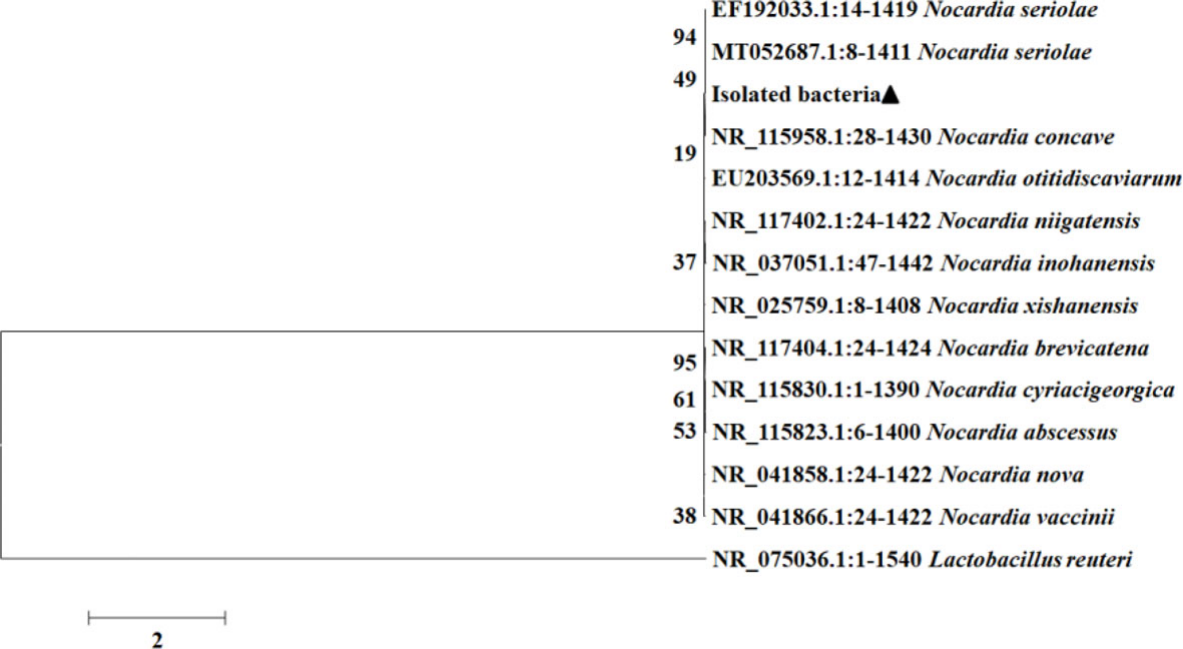
Phylogenetic analysis of 16S rDNA from the isolated bacteria, N. seriolae and other Nocardia sp. The phylogenetic tree was constructed using MEGA 6.0 with the Mmaximum likelihood method. Numbers of each node indicate the percentage of bootstrapping in 100 replications.

After 7 d of BHI culture, the pathogenic bacteria formed white sand granular colonies of different sizes with rough surface and irregular edges (**Supplementary Figure 1**). A single band (1600 bp) could be amplified by PCR with 16S rDNA primers. The 16S rDNA sequences of isolated bacteria were 99% consistent with that of *N. seriolae* (**Supplementary Figure 2**). Phylogenetic tree analysis showed that the bacteria isolated in this study were clustered with *N. seriolae* (**Figure 2**). Furthermore, the isolated bacteria were used to inject the healthy hybrid snakeheads intraperitoneally. The results showed that the hybrid snakeheads (n=10) injected with the bacteria got infected within 48 h and died in succession within 72-144 h. The clinical symptoms of the dead fish were consistent with those of the naturally diseased fish in the pond. The symptoms of ascites were not obvious. However, there was no morbidity or death in the control group.

### FISH Results

**Figure 3:**
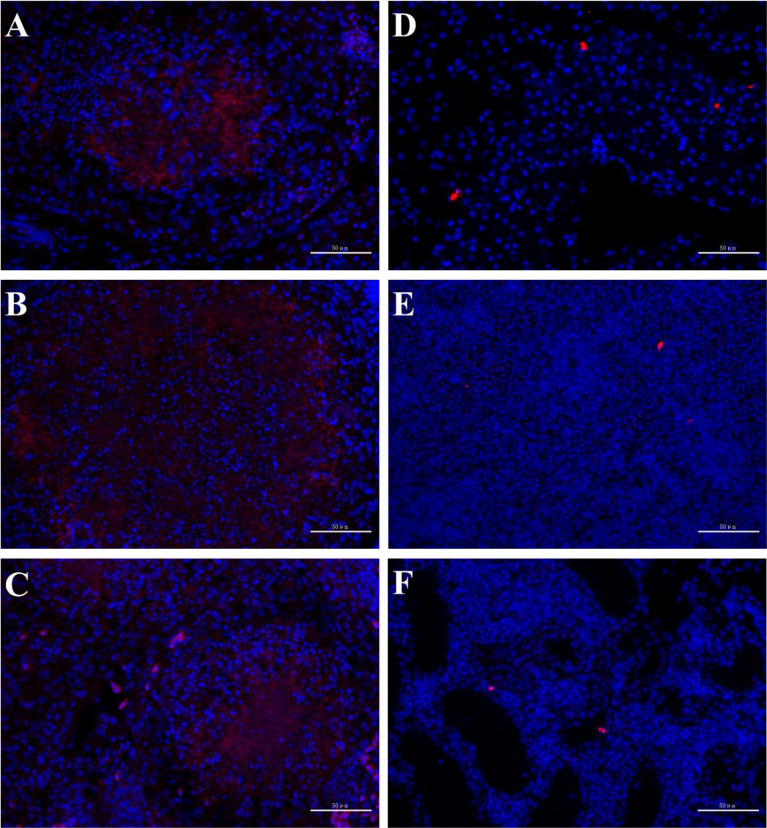
FISH of hybrid snakehead. The probe had strong hybridization signals in the liver **(A)**, spleen **(B)** and kidney **(C)** of diseased fish, but weak hybridization signals in the liver **(D)**, spleen **(E)** and kidney **(F)** of healthy fish. Scale bar = 50 mm.

In order to observe the infection of pathogenic bacteria in immune organs, FISH was conducted on tissue sections of the liver, spleen, and kidney of both diseased and healthy individuals. The results showed that there were strong positive signals in nodule sites of liver, spleen, and kidney of diseased individuals, while there were only weak hybridization signals in healthy hybrid snakeheads (**Figure 3**).

### Transcriptome Assembly and Functional Annotation

**Figure 4:**
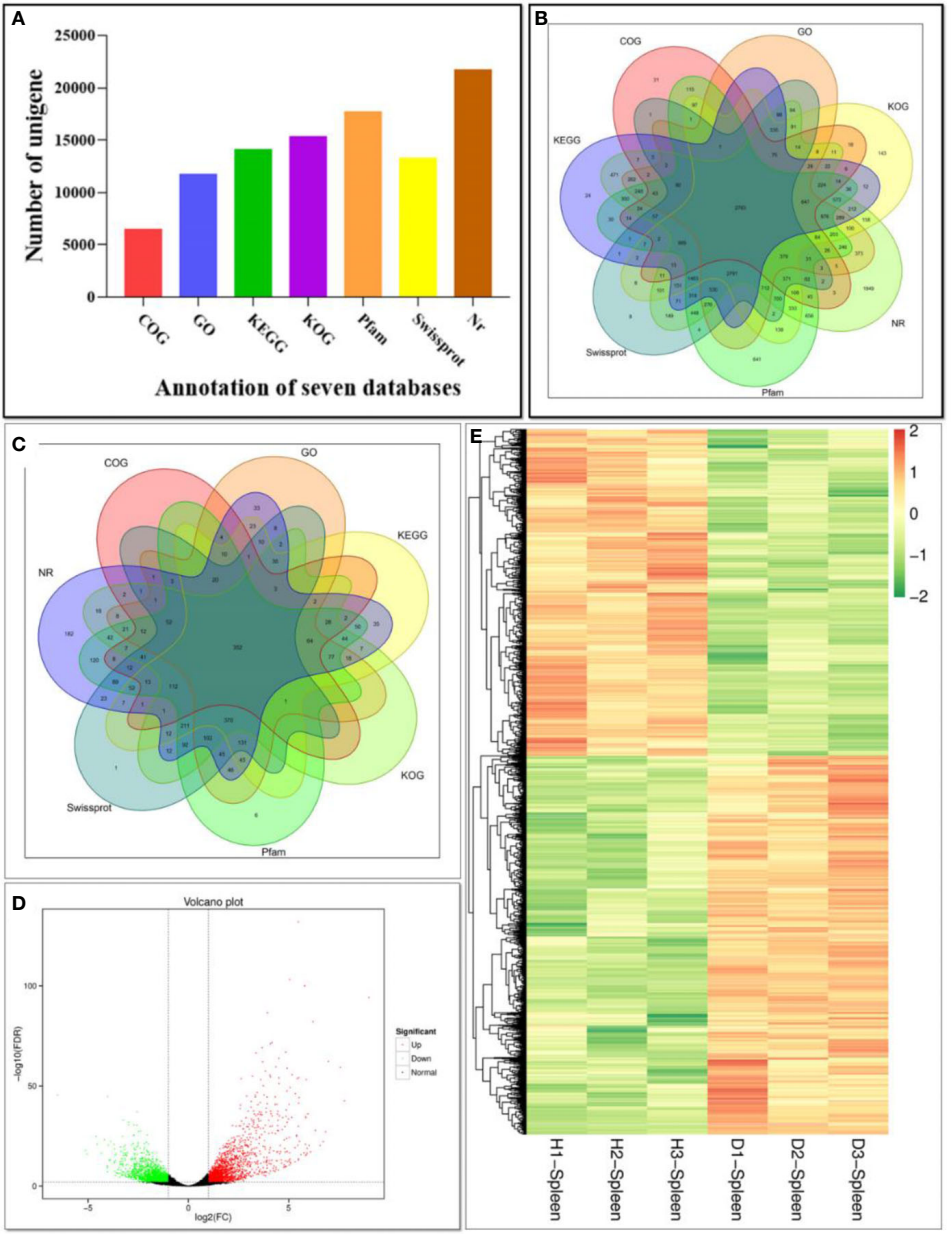
Function annotation of unigenes based on BLAST against seven databases. **(A)** Numbers of unigenes annotated in seven databases. The X-axis represents different databases. The Y-axis corresponds to the number of unigenes.; **(B)** Venn diagram of unigenes annotated in seven databases; **(C)** Venn diagram of DEGs annotated in seven databases; **(D)** Volcano plot of DEGs comparing the diseased fish and healthy fish. The X-axis represents fold change between diseased and healthy groups. The Y-axis indicates the statistical significance of the difference of gene expression. Each dot in the diagram represents a gene, and the black dots represent no significant changes in expressed genes, while the red dots and the green dots represent up- and down- regulated in diseased fish, respectively. **(E)** Heat map of DEGs comparing the diseased fish and healthy fish. H1, H2 and H3 represent three samples of healthy individuals, and the D1, D2 and D3 represent three samples of diseased individuals. Each row represents a gene. Gene expression levels are shown with different colors. Negative and positive numbers indicate down- and up-regulation, respectively.

A total of 41.44 Gb clean data was obtained, and the Q30 of each sample was ≥ 93.84% (**Supplementary Table 2**). The assembled transcriptome had 118,340 contigs with an N50 length of 3,077 bp and 55,203 unique gene transcripts (or unigenes) with an N50 length of 2,762 bp (**Supplementary Table 3**). The 55,203 unigenes were searched in NR, Swiss-Prot, COG, KOG, Pfam, GO and KEGG databases. A total of 24,498 unigenes were annotated in at least one database, including 21,766 in NR, 15,359 in KOG, 14,161 in KEGG, 6,496 in COG, 17,748 in Pfam, 13,273 in Swissprot and 11,749 in GO (**Figures 4A, B**).

### Identification of Differentially Expressed Genes (DEGs) and Enrichment Analysis

**Figure 5:**
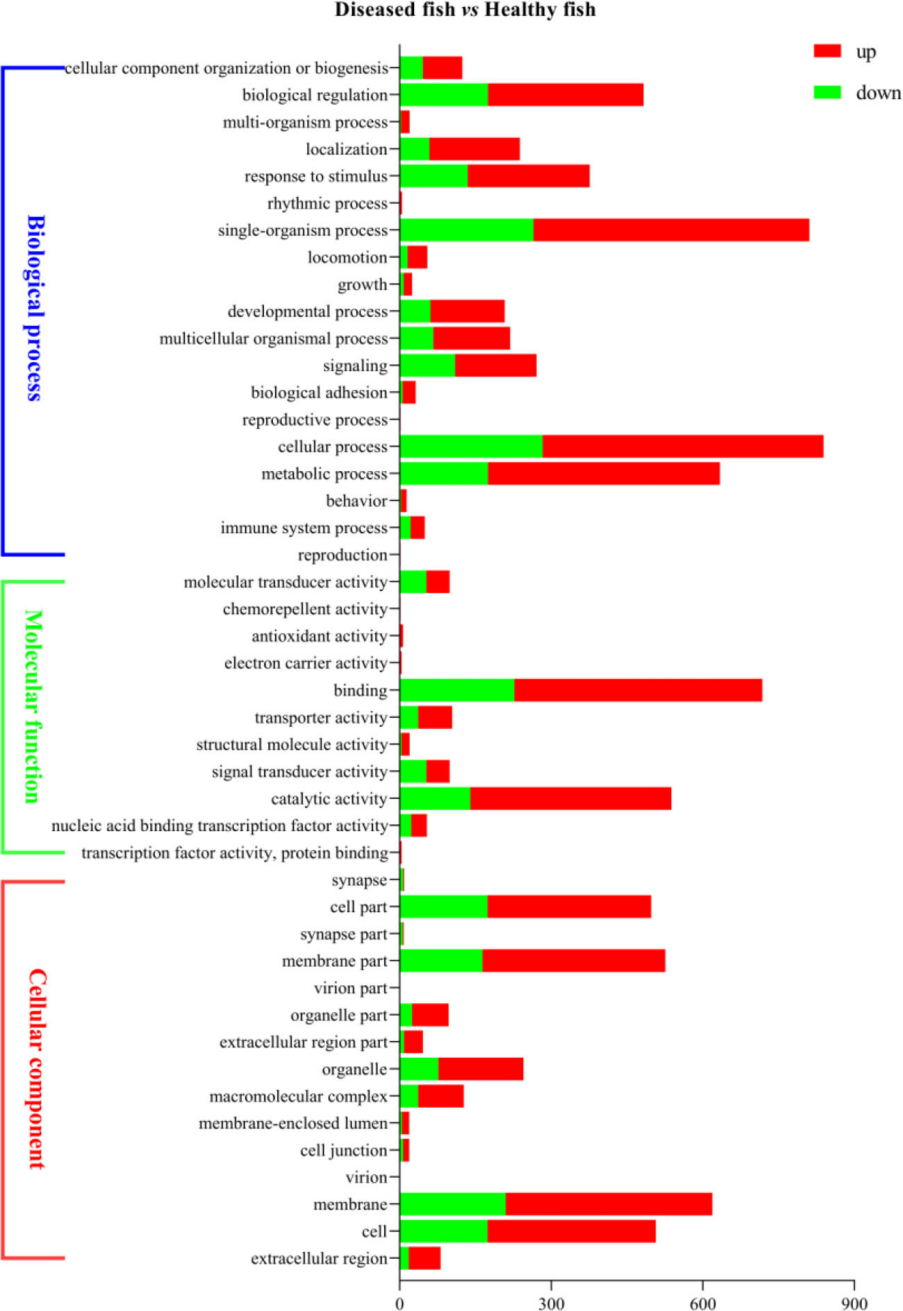
Bar graph description of Gene Ontology (GO) enrichment of DEGs. The X-axis corresponds to the number of DEGs. The Y-axis represents various GO terms. The healthy group was used as the control group, and the green bar and red bar represent down- and up-regulated DEGs, respectively.

**Figure 6:**
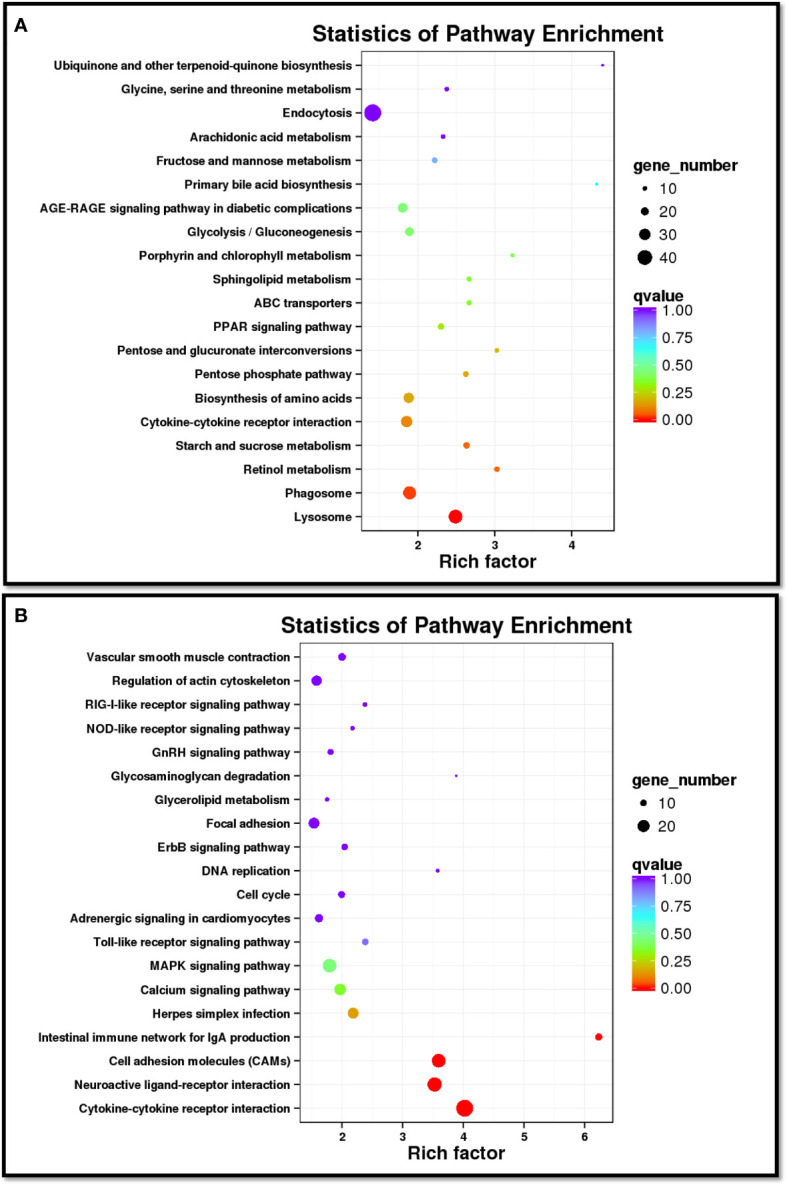
Scatter diagram of the top 20 enriched KEGG pathways for up-regulated **(A)** and down-regulated **(B)** DEGs in diseased spleen sample. The X-axis corresponds to the rich factor of a pathway, and the rich factor is the ratio of DEGs to all the genes in a specific pathway. The Y-axis represents different pathways. The magnitude of the dots displays gene number from 10 to 40 **(A)** or 10 to 20 **(B)**, and the q-value is described by the color classification.

In order to analyze the similarities and differences in spleen samples between healthy and diseased fish, PCA was conducted. The spleen samples from diseased fish replicates were clustered closely in a region of the PCA plot, and healthy individuals were scattered in another region (**Supplementary Figure 3**). A total of 3,512 unigenes were differentially expressed between healthy and diseased fish spleen (|log2fold change| ≥1, FDR ≤0.01). Of these, 1,886 unigenes were up-regulated in the spleen of diseased fish (**Figures 4C**–**E**). A total of 1,494 DEGs were classified into three GO categories, including the biological process, molecular function, and cellular component (**Figure 5**). Most of the DEGs in the category of biological processes were involved in cellular process (839 unigenes), single-organism process (811 unigenes) and metabolic process (634 unigenes). DEGs in the molecular function category were mainly involved in binding (718 unigenes) and catalytic activity (538 unigenes), while DEGs in the category of cellular components were mainly related to membrane (619 unigenes) and membrane part (526 unigenes) (**Figure 5**). A total of 1,731 DEGs were annotated to 170 signaling pathways in the KEGG database (**Supplementary Table 4**). Further analysis showed that the up-regulated DEGs in diseased fish were significantly enriched in lysosome (ko04142) and phagosome (ko04145) (**Figure 6A**), while the down-regulated DEGs were significantly enriched in cytokine- cytokine receptor interaction (ko04060), neuroactive ligand-receptor interaction (ko04080), cell adhesion molecules (CAMs) (ko04514) and intestinal immune network for IgA production (ko04672) (**Figure 6B**).

### qPCR Validation and Tissue Expression Analysis

**Figure 7:**
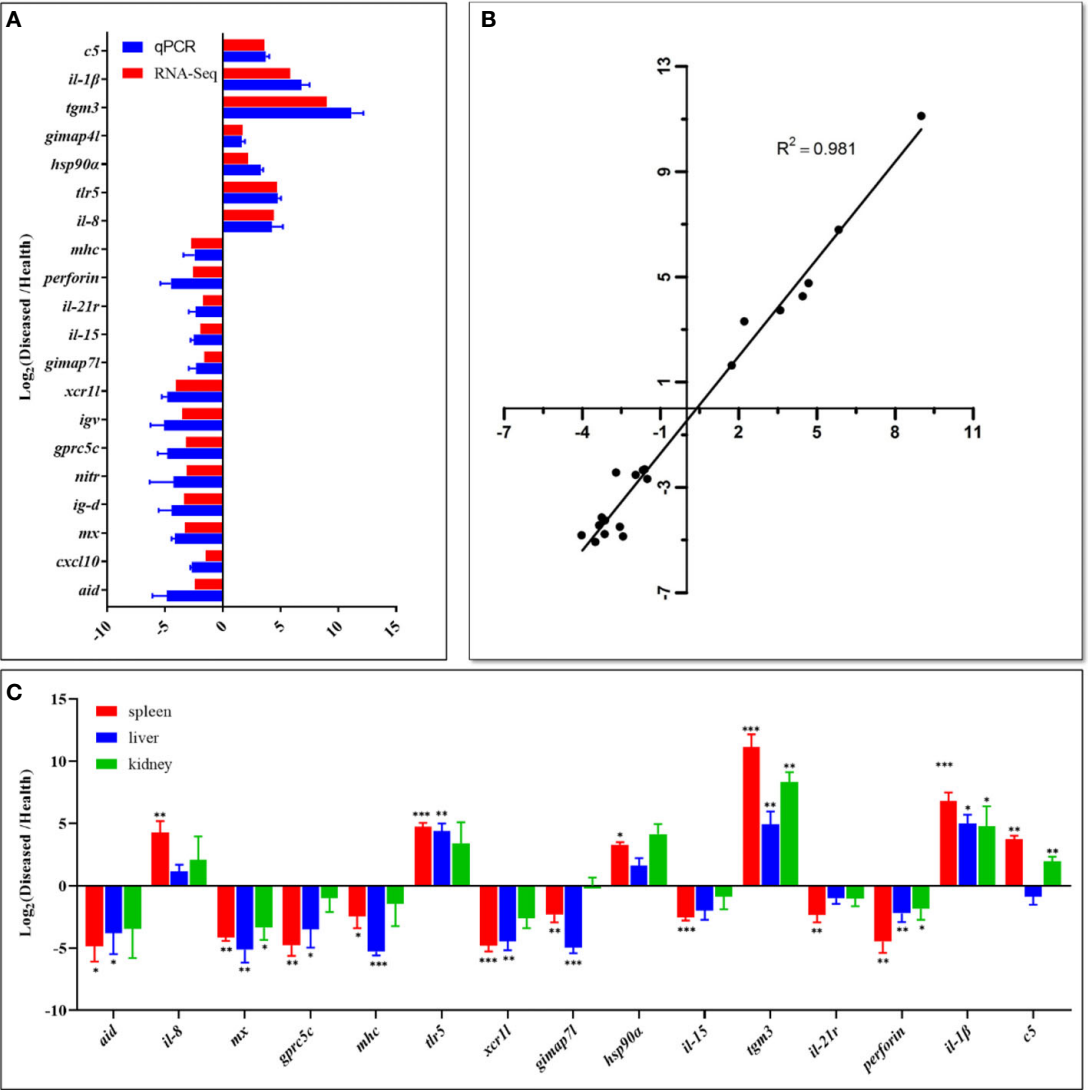
Comparison of gene expression data between RNA-Seq and qPCR. **(A)** The relative expression levels of twenty DEGs detected by qPCR were compared with that of RNA-Seq. The X-axis represents the value of RNA‐Seq data and qPCR data, and the Y-axis represents the gene name. RNA-Seq data used log_2_fpkm values of diseased and healthy fish samples, and qPCR data are presented as mean ± standard deviation (n=3). The p value of all genes in qPCR was less than 0.01. **(B)** Correlation of qPCR and RNA-Seq data. The X-axis represents the RNA-Seq data, and the Y-axis represents the qPCR data. The Pearson product-moment correlation coefficient (R^2^) of each plot was shown as an indication of positive linear correlation between the two methods. **(C)** The expression patterns of fifteen selected DEGs in the spleen, liver, and kidney. qPCR data are presented as mean ± SD (n=3). The X-axis represents the gene name, and the Y-axis represents the value of qPCR data. Statistically significant differences in gene expression levels between healthy and diseased fish in each tissue type are marked with asterisks (* means p < 0.05, ** means p < 0.01, *** means p < 0.001). The raw data (Ct values) of qPCR is in **Supplementary Table 6**.

The qPCR results showed that the expression levels of the examined genes were consistent with the RNA-Seq analysis, implying that the RNA-Seq data were reliable (**Figures 7A, B**). In addition, the transcription levels of fifteen DEGs were investigated by qPCR in the liver and kidney of diseased and healthy fish. The results showed that the overall expression trends of these genes were similar in the spleen, liver, and kidney (**Figure 7C**). However, interferon-induced GTP-binding protein Mx (*mx*)*, perforin*, protein-glutamine gamma-glutamyltransferase E (*tgm3*) and interleukin-1 beta (*il-1β*) genes showed significantly different expression levels in all types of tissues between diseased and healthy fish, while single-stranded DNA cytosine deaminase (*aid*), G-protein coupled receptor family C group 5 member C (*gprc5c*), major histocompatibility complex (*mhc*), toll-like receptor 5 (*tlr5*) and GTPase IMAP family member 7-like (*gimap*7*l*) genes were differentially expressed in the spleen and liver (**Figure 7C**).

## Discussion

In the present study, some diseased hybrid snakeheads from artificial breeding ponds were collected for histological and transcriptomics analyses. Diseased fish showed some symptoms, including no food intake, reduced vitality, scale injury, anal swelling, ascites, and multiple white nodules on the surface of liver, kidney, and spleen (**Figure 1**). These symptoms are consistent with fish *Nocardia*, and the pathogen was presumed to be *Nocardia* spp (5, 6, 17, 26, 29, 30). The growth rate and colony structure of the pathogen were similar to that of *Nocardia* spp. (**Supplementary Figure 1**) (6, 16). The bacteria isolated in this study were identified as *N. seriolae* (**Figure 2**, **Supplementary Figure 2**) by 16S rDNA sequencing and phylogenetic analysis. The clinical symptoms of healthy hybrid snakeheads challenged with the isolated bacteria were the same as those observed in naturally infected fish, confirming that *N. seriolae* was the cause of this disease in cultured hybrid snakeheads. The phenomenon of hybrid snakehead ascites was not obvious after *N. seriolae* injection, as the injection concentration of the pathogen was probably too high and the hybrid snakehead died within a short period (72-144 h).

In the breeding process, *N. seriolae* may infect immunocompromised hybrid snakeheads through the feeds, gills, and wounds, resulting in a chronic systemic granulomatous disease and leading to ascite symptoms (5). Intraperitoneal injection of *N. seriolae* resulted in acute symptoms and mortality of more than 60% in infected fish. *N. seriolae* infection is a long-term process in aquaculture ponds, and the mortality rate is about 30% (5, 6, 17, 31, 32). FISH analysis also showed that *N. seriolae* aggregated locally in the liver, spleen and kidney of the diseased fish and destroyed the corresponding cell structure, resulting in cell necrosis (**Figure 3**).

In order to dissect the immune response of cultured hybrid snakeheads to *N. seriolae* infection, spleen transcriptomes were analyzed. A total of 55,203 unigenes with a N50 length of 2,762 bp were assembled. Of these, 24,498 (44.38% of 55,203) unigenes were annotated. The annotation rate of hybrid snakehead is higher than smooth tongue sole (*Cynoglossus semilaevis*) (41.51% of 89,648 unigenes), but lower than largemouth bass (78.76% of 47,881) (24) and Japanese seabass (*Lateolabrax maculatus*) (82.32% of 86,268) (33), which may be due to the complex genetic makeup of this hybrid species, lack of reference genomic data in the database (34, 35), presence of unannotated unigenes containing 3’ or 5’ untranslated regions and non-coding RNAs, or occurrence of new unigenes and short sequences containing unknown protein domains (36).

According to GO analysis, the number of up-regulated DEGs was more than that of down-regulated genes in the three GO categories in infected fish (**Figure 5**). By contrast, the number of down-regulated genes was more than that of up-regulated genes in the sub-categories of molecular transducer activity and signal transducer activity. KEGG analysis showed that a total of 42 DEGs (16 up-regulated and 28 down-regulated in diseased fish) were enriched in five immune system pathways (**Supplementary Table 5**), including “Intestinal immune network for IgA production”, “Toll-like receptor signaling pathway”, “NOD-like receptor signaling pathway”, “RIG-I-like receptor signaling pathway” and “Cytosolic DNA-sensing pathway”. These immune system pathways were also altered by pathogenic infection in other cultured fish. Transcriptome analysis of the largemouth bass showed that the immune system pathways (5, 24), including “Intestinal immune network for IgA production”, “Toll-like receptor signaling pathway”, “NOD-like receptor signaling pathway” and “Cytosolic DNA-sensing pathway”, were enriched in the spleen cells after *Edwardiana* infection (37). “Intestinal immune network for IgA production”, “Toll-like receptor signaling pathway”, “NOD-like receptor signaling pathway” and “RIG-I-like receptor signaling pathway” were enriched in the spleen and head kidney transcriptomes of Nile tilapia (*Oreochromis niloticus*) after *Streptococcus agalactiae* infection (38). Half-smooth tongue sole infected by *Vibrio anguillarum*, and the “Toll-like receptor signaling pathway”, “NOD-like receptor signaling pathway” and “Cytosolic DNA-sensing pathway” were enriched in the spleen of fish (39). These findings indicated that these primary host immune system pathways were conserved in teleosts, which are activated to defend against pathogenic invasion (40–43).

Phagocytosis is the process by which cells engulf large particles. It is a central mechanism in inflammation and defense against infectious agents (44, 45). A phagosome is formed when the specific receptors on the phagocyte surface recognize ligands on the particle surface (45). Lysosomes are membrane-delimited organelles in animal cells serving as the cell’s main digestive compartment to extracellular materials that have been internalized by endocytosis and intracellular components that have been sequestered by autophagy (46). Phagosomes fuse with lysosomes to generate phagolysosomes, which play a crucial role in enzymatic digestion of the internalized contents into component parts (47, 48). In this study, up-regulated DEGs in diseased fish were significantly enriched in “Phagosomes” pathway (**Supplementary Figure 4**) and “Lysosomes” pathway (**Supplementary Figure 5**), indicating that the functions of phagosome and lysosome are affected in the hybrid snakehead with nocardiosis. Lysosome-associated membrane proteins 1 and 2 (LAMP-1 and LAMP-2) are delivered to phagosomes during the maturation process (48). Calreticulin and calnexin are Ca^2+^-binding proteins and play important roles in phagocytosis (49, 50). Cathepsin S (*cats*) plays an important role in lysosome, phagosome, apoptosis and antigen processing and presentation pathway (51). *Calnexin* and *cats* were up-regulated in the spleen of diseased hybrid snakehead, indicating that the diseased fish had undergone phagocytosis and lysosomal enzymatic hydrolysis. However, natural resistance-associated macrophage protein 1 (*nramp*1), which is a lysosomal marker and plays an important role in the immune response of teleost to pathogenic bacteria (52–54), was induced in diseased fish spleen. Taken together, the Phagosome and Lysosome-related DEGs in the spleen of diseased fish suggested that the Phagocytosis and Lysosomal activities of fish were prominently triggered by the *N. seriolae* infection (55).

Cytokines are soluble extracellular proteins or glycoproteins that are crucial intercellular regulators and mobilizers of cells engaged in innate immunity and play important roles in adaptive inflammatory host defenses and restoration of homeostasis (56–58). Covello et al. found that expression levels of three pro-inflammatory cytokines in striped trumpeter (*Latrislineata forster*), including TNF-α, IL-1β and IL-8, were significantly changed in response to infection by *Chondracanthus goldsmidi* (59). Tanekhy et al. challenged Japanese flounder with *N. seriolae* and the expression levels of *tnf*α and *il-1β*were significantly increased in the spleen and head kidney of fish and then rapidly declined to pre-infection levels (31). Byadgi et al. conducted comparative transcriptome analysis of the spleen of largemouth bass before and after *N. seriolae* infection, and found that different cytokines and cytokine receptor families were up-regulated (24). Li and Zhang found that ILs, and TNFs were down-regulated in Japanese flounder after injecting attenuated *Edwardsiella tarda* (55). In the present study, a total of 59 DEGs were involved in cytokine-cytokine receptor interaction signaling pathways (**Supplementary Figure 6**), and 30 DEGs of these were up-regulated in diseased fish, including C-C chemokine ligand 25 (*ccl*25), C-C chemokine receptor type 4 (*ccr*4), interleukin 11 (*il*11), tumor necrosis factor (*tnf*), activin receptor type-1B (*acvr*1*b*), activin receptor type-II B (*acvr*2*b*), etc. The other 29 DEGs were down-regulated in diseased fish, including C-X-C motif chemokine 10 (*cxcl*10), C-X-C motif chemokine receptor 3 (*cxcr*3), C-C chemokine receptor type 2 (*ccr*2), C-C chemokine receptor type 5 (*ccr*5), C-C chemokine receptor type 7 (*ccr*7), C-C chemokine receptor type 9 (*ccr*9), interleukin 12 (*il*12), interleukin 15 (*il*15), tumor necrosis factor receptor superfamily member 13B (*tnfsf*13b), tumor necrosis factor receptor superfamily member 11 B (*sf*11*b*), etc. These results indicated that *N. seriolae* infection disrupted the function of cytokine-cytokine receptor interaction in the spleen of hybrid snakeheads (58, 60).

Cell adhesion molecules (CAMs) are glycoproteins expressed on the cell surface and play a critical role in hemostasis, immune response, and inflammation (61). Membrane proteins mediate immune cell-cell interactions such as antigen recognition, costimulation and cellular adhesion (61). Identification of pathogens is the initiation of an immune response (62). In this study, 41 CAMs were significantly enriched. Of these, *mhc*, class I (*mhc*1) major histocompatibility complex, class II (*mhc*2), programmed cell death 1 ligand 1 (*pdl*1), T-cell surface glycoprotein CD8 beta chain (*cd*8), CD226 antigen (*cd*226) and myelin-associated glycoprotein (*mag*) were inhibited in the diseased fish spleen, while CD99 antigen (cd99), cadherin 5 (*cdh*5), junctional adhesion molecule 1 (*jam*1), claudin (*cldn*), and poliovirus receptor-related protein 1 (*pvrl*1) were activated (**Supplementary Figure 7**). These results suggested that the diseased fish might experience disrupted cell-cell interactions (24, 37, 55).

In addition, the liver and kidney of fish are the target organs of *N. seriolae* infection and play essential roles in the immune response of fish (6, 8, 17). In this study, the clinical symptoms of the liver and kidney of diseased fish were similar to those of the spleen (**Figures 1**, **3**). The expression patterns of 15 DEGs in the spleen were comparable to those in the liver and kidney (**Figure 7C**). These results suggested that *N. seriolae* also influenced the immune responses in the liver and kidney of hybrid snakeheads. *N. seriolae* could significantly change the expression levels of *mx, perforin, tgm3, il-*1*β* and other genes in the kidney, liver, and spleen of diseased hybrid snakeheads, indicating that these genes have the potential to be developed as biomarkers for fish nocardiosis.

## Conclusion

In this study, hybrid snakeheads with symptoms of nocardiosis disease in an aquaculture pond were analyzed, and *N. seriolae* was identified as the causative agent of nocardiosis by 16S rDNA sequencing and artificial infection experiment. The transcriptome profiles of spleen from diseased and healthy hybrid snakeheads were investigated using RNA-seq. A total of 3,512 unigenes were differentially expressed in the diseased fish, and 1,886 of these were up-regulated. Multiple immune-related genes and pathways were identified by GO and KEGG analyses, and several biological processes including phagocytosis, cytokine-cytokine receptor interaction, and immune cell-cell interactions are likely to play essential roles in fish immune response to *N. seriolae* infection. These results provide necessary information for further research on the immune defense mechanisms of hybrid snakehead to *N. seriolae*, which will help with biomarker screening of fish *Nocardia* spp. and the breeding program of nocardiosis-resistant fish.

## Data Availability Statement

The datasets presented in this study can be found in online repositories. The names of the repository/repositories and accession number(s) can be found in the article/**Supplementary Material**.

## Ethics Statement

The animal study was reviewed and approved by Animal Research and Ethics Committee of Guangdong Ocean University (201903003).

## Author Contributions

NZ, ZD, and WW contributed to the conception and design of the study. NZ, ZD, HZ, and WW contributed to the writing, review, and/or revision of the manuscript. ZD and WW provided analytical technical support. All authors contributed to the article and approved the submitted version.

## Funding

This work was supported by the Guangdong Basic and Applied Basic Research Foundation (#2020A1515110086, #2018A030310049); the Natural Science Foundation of China (^#^32102796); Special innovation projects of colleges and universities in Guangdong province (#2018KTSCX087, #2018KQNCX110); Nanhai Scholar Project of GDOU (#QNXZ201807, #QNXZ 201903); Guangxi Key Laboratory of Beibu Gulf Marine Biodiversity Conservation (2019KB06).

## Conflict of Interest

Author HZ is employed by Zhongshan Ronghai aquaculture Co. Ltd.

The remaining authors declare that the research was conducted in the absence of any commercial or financial relationships that could be construed as a potential conflict of interest.

## Publisher’s Note

All claims expressed in this article are solely those of the authors and do not necessarily represent those of their affiliated organizations, or those of the publisher, the editors and the reviewers. Any product that may be evaluated in this article, or claim that may be made by its manufacturer, is not guaranteed or endorsed by the publisher.

## Supplementary Material

Supplementary Table 6 The raw data (Ct values) of qPCR.
