# Supplementary material for: Molecular Identification of Nocardia seriolae and Comparative Analysis of Spleen Transcriptomes of Hybrid Snakehead (Channa maculata Female × Channa argus Male) With Nocardiosis Disease
Source: Front Immunol. 2022 Jan 27;13:778915. doi: 10.3389/fimmu.2022.778915 (PMC8828968; doi:10.3389/fimmu.2022.778915)
Supplement: Supplementary file 2 [file DataSheet_1.docx]

Supplementary Material


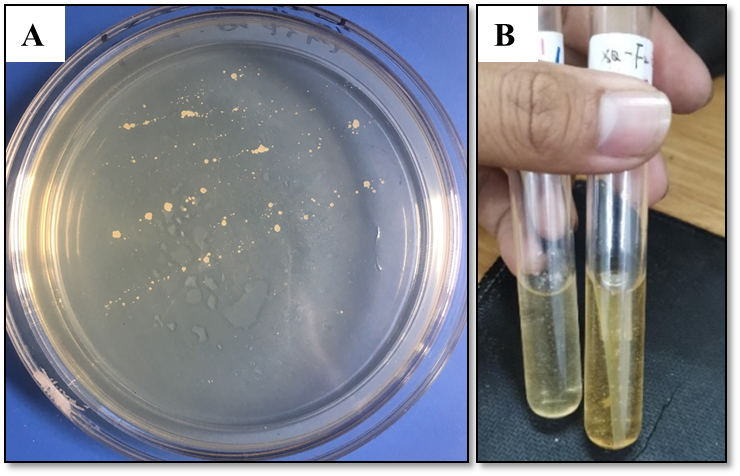


**Supplementary Figure 1.** Isolation and culture of *N. seriolae.* (A) Solid culture colony morphology of *N. seriolae;* (B) *N. seriolae* in liquid culture.


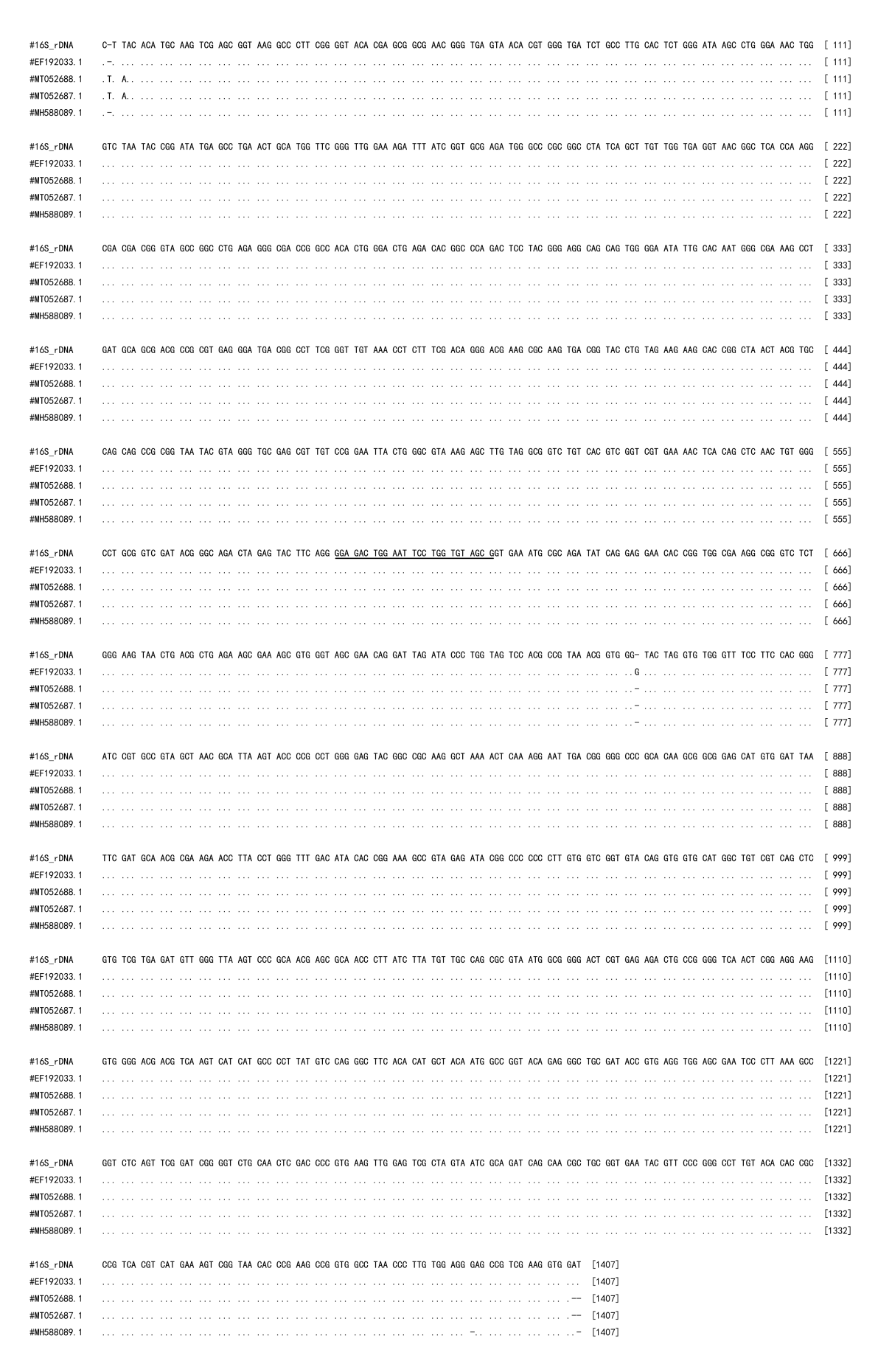


**Supplementary Figure 2.** Multiple sequence alignment of 16S rDNA from hybrid snakehead pathogenic bacteria and other species. “.”: The same amino acids. “-”: Missing sequence. The underscore indicates the sequence of probes for FISH


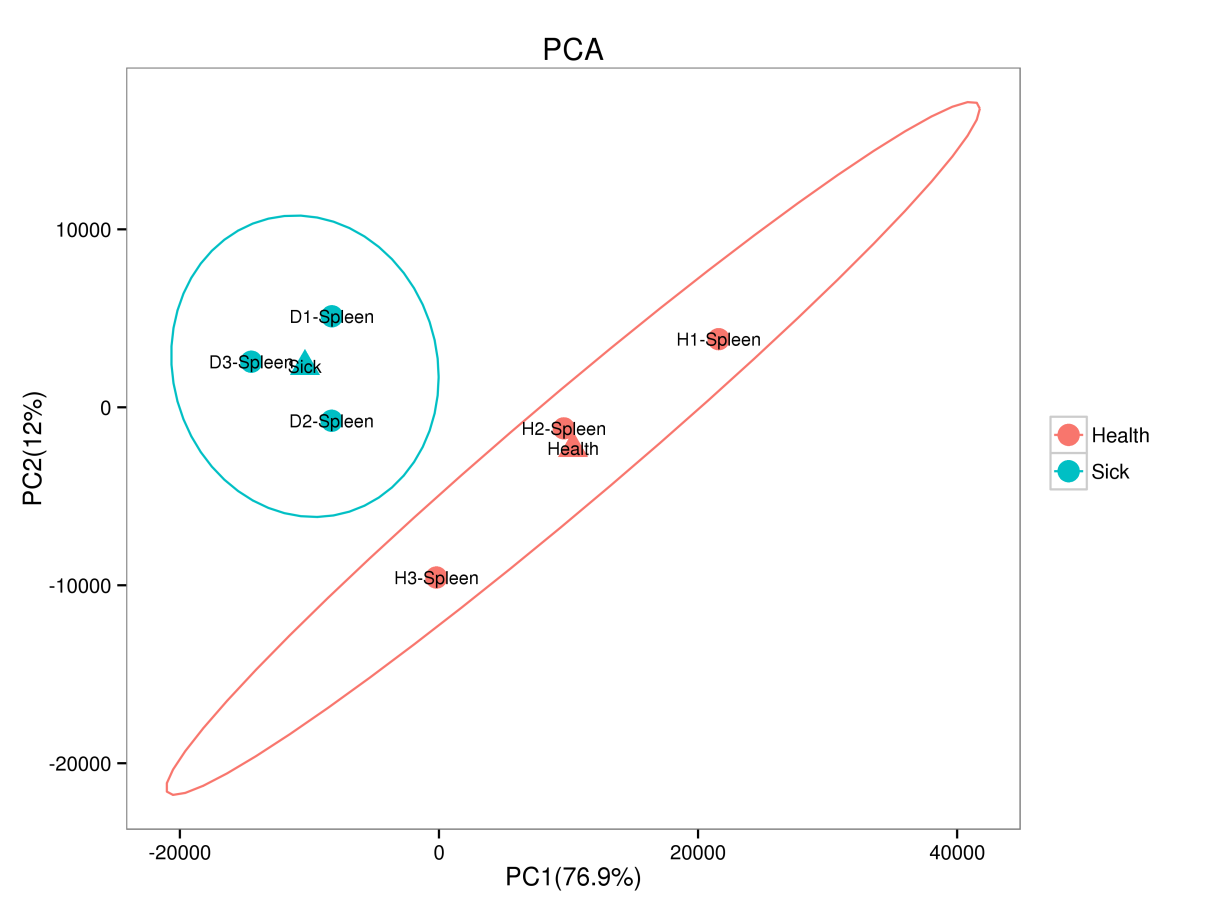


**Supplementary Figure 3.** PCA plot of sequencing samples. The green dots represent the spleen samples of diseased hybrid snakehead, and the red dots represent the spleen samples of healthy fish.


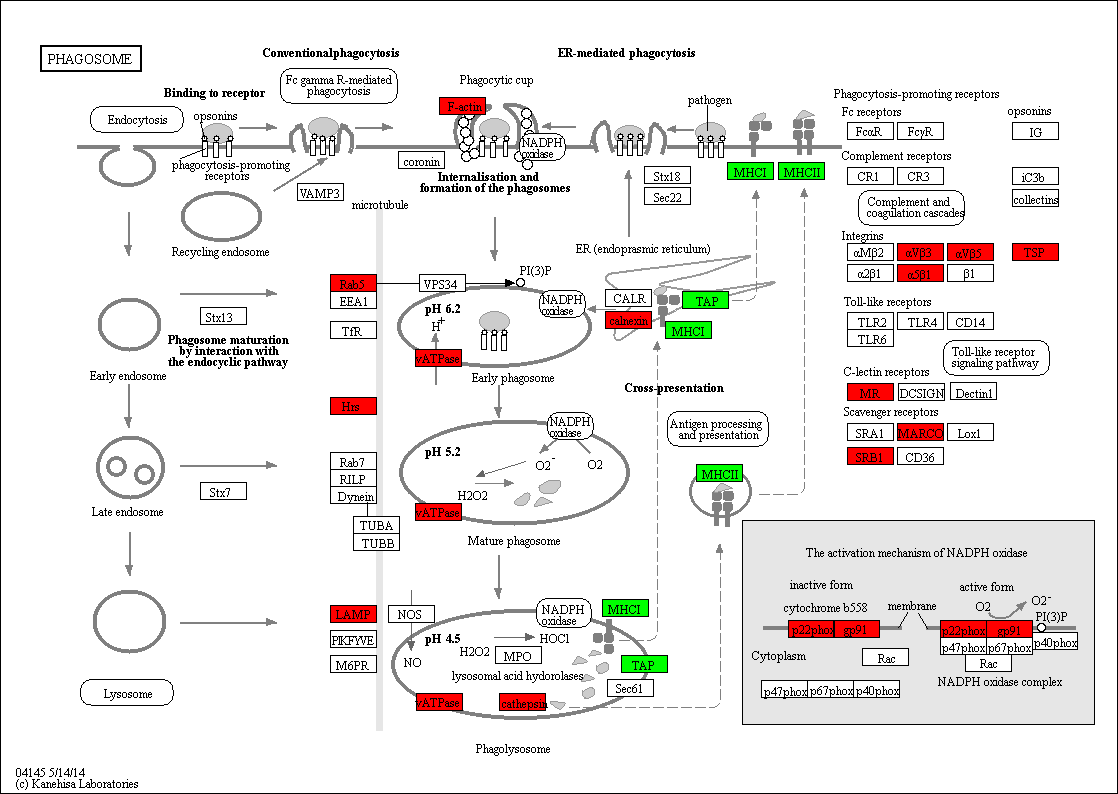


**Supplementary Figure 4.** The “Phagosome” pathway. The red boxes represent up-regulated genes in the spleen of diseased fish, while the green boxes represent down-regulated genes in the spleen of diseased fish.


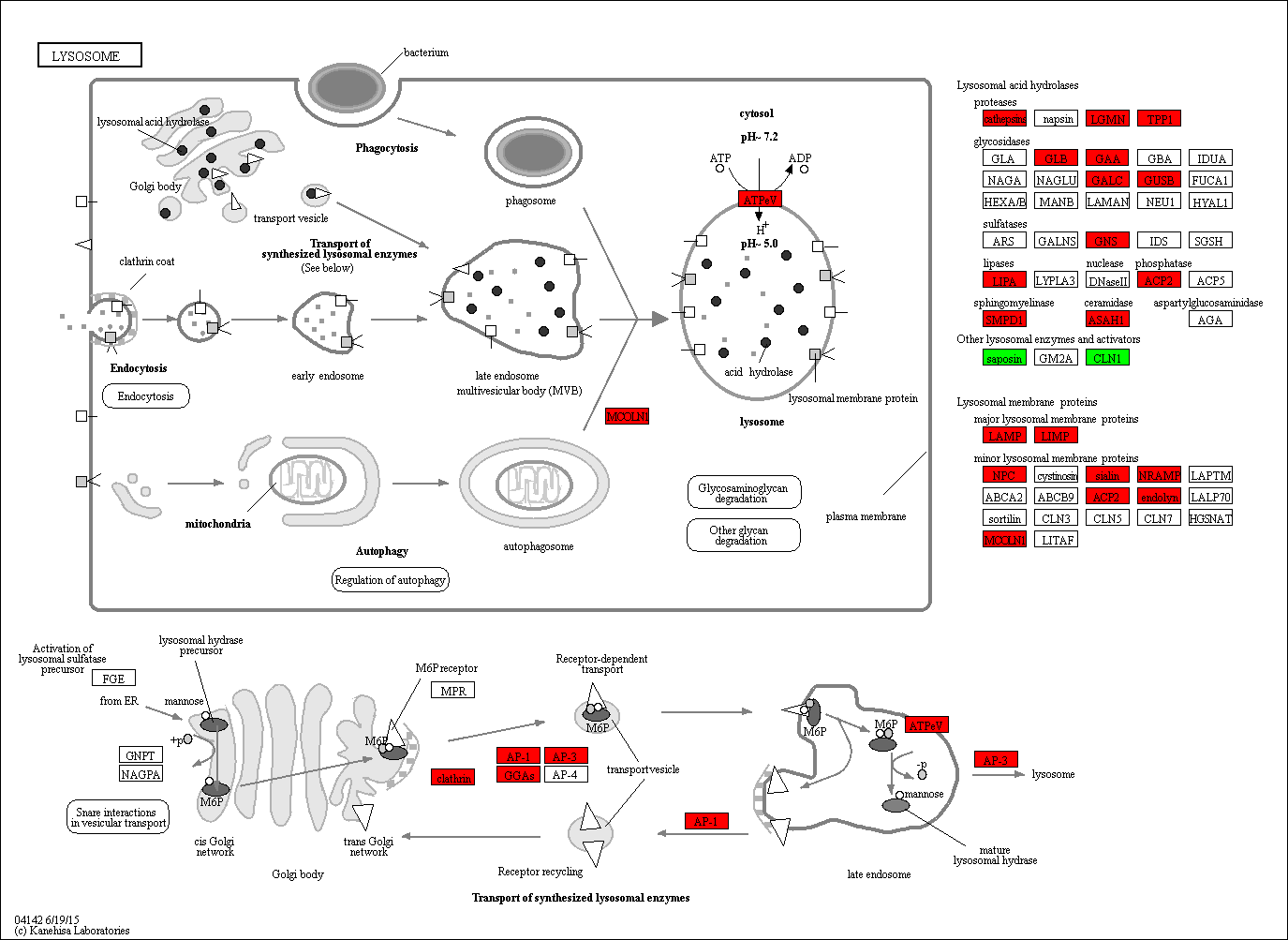


**Supplementary Figure 5.** The “Lysosome” pathway. The red boxes represent up-regulated genes in the spleen of diseased fish, while the green boxes represent down-regulated genes in the spleen of diseased fish.


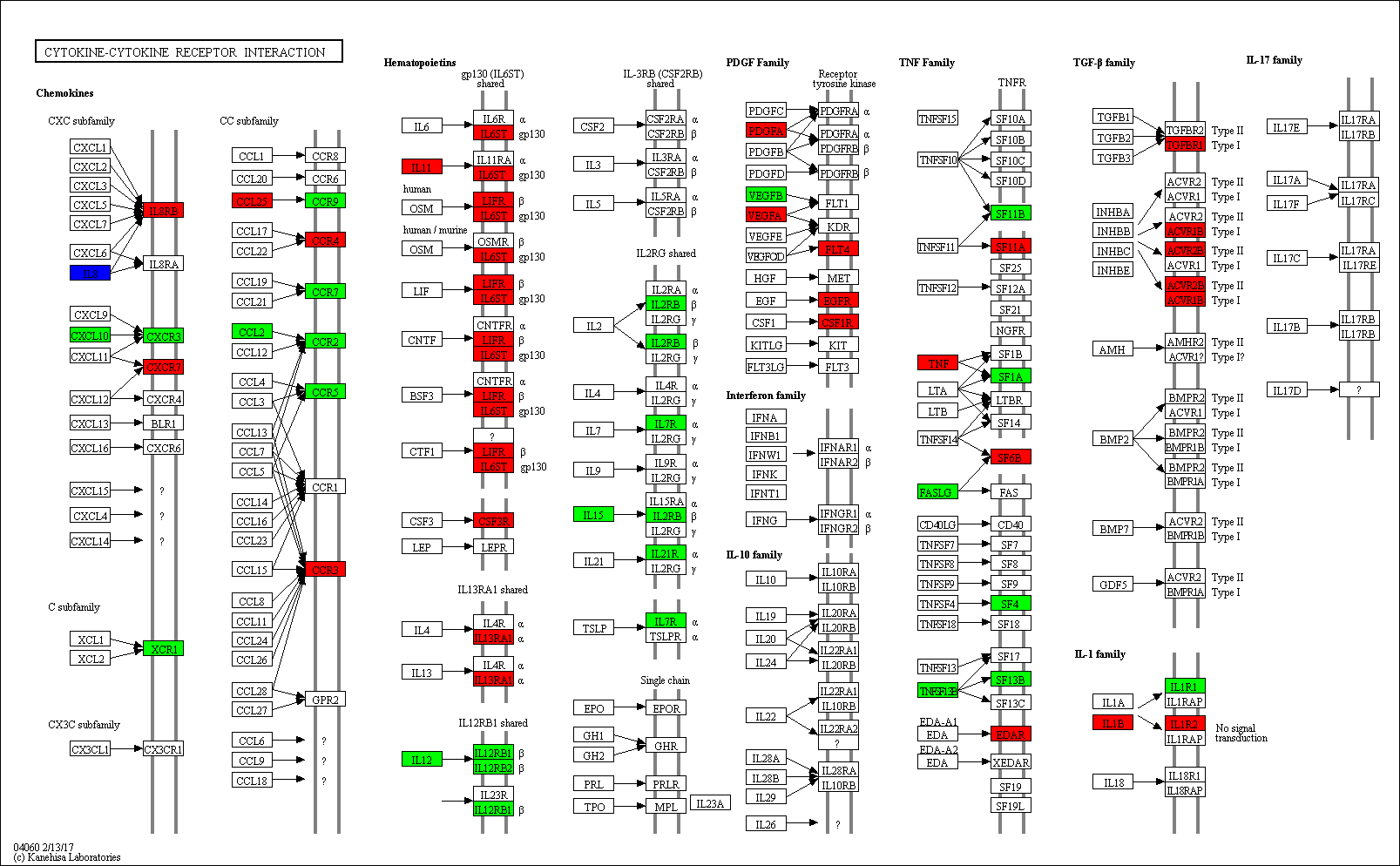


**Supplementary Figure 6.** The “Cytokine- cytokine receptor interaction” pathway. The red boxes represent up-regulated genes in the spleen of diseased fish, while the green boxes represent down-regulated genes in the spleen of diseased fish.


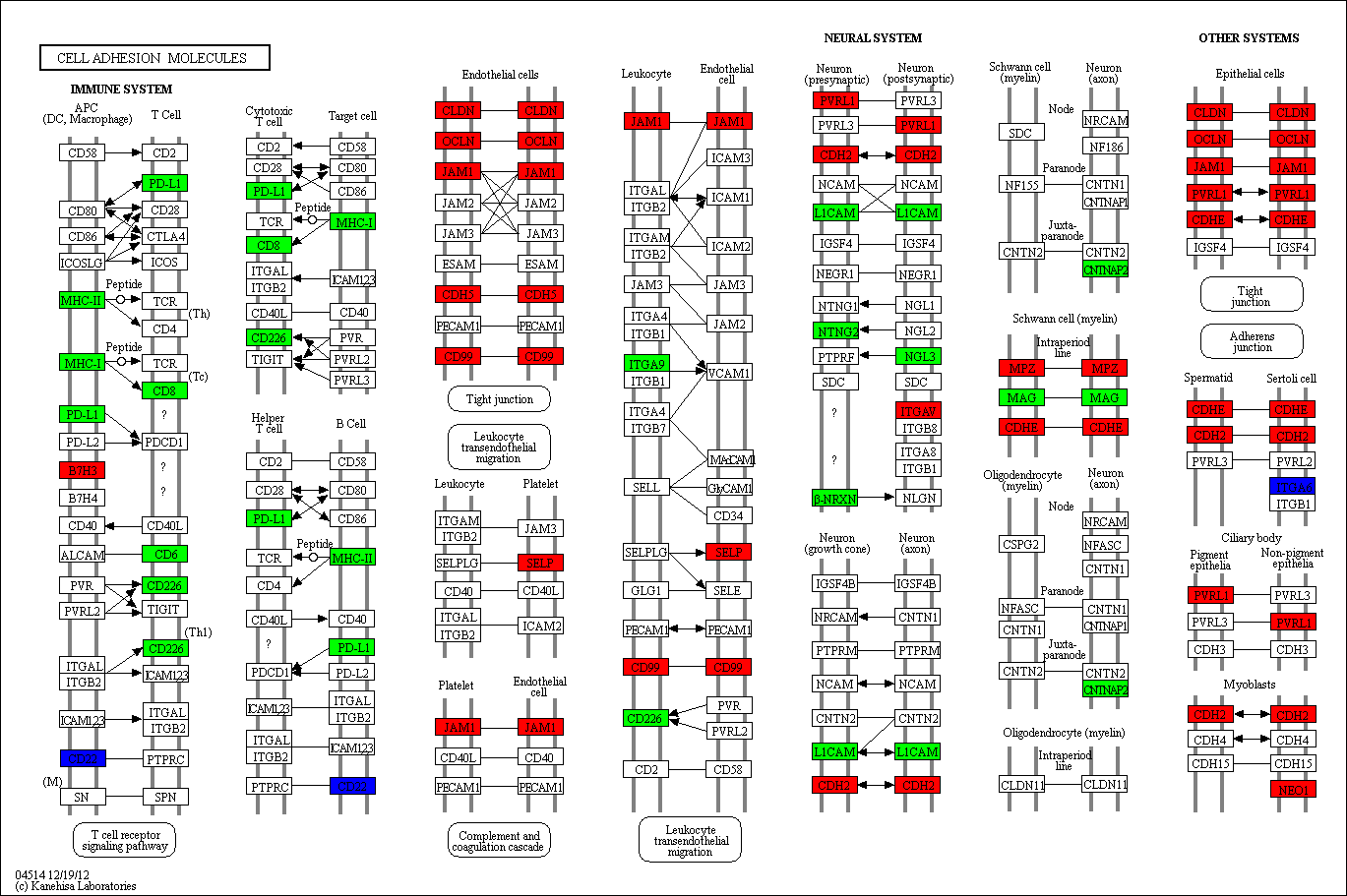


**Supplementary Figure 7.** Cell adhesion molecules (CAMs). The red boxes represent up-regulated genes in the spleen of diseased fish, while the green boxes represent down-regulated genes in the spleen of diseased fish.

**Supplementary Table 1** Primers and their sequences used in this study

| Gene name | Sequence 5-3 | Purpose |
| --- | --- | --- |
| *il-*8 | F: GAGCCTAAAGATTCATAAGAGGGC | qPCR |
|  | R: CAGACTCATACCTTCACTGATGGC |  |
| *mx* | F: GAAACCCTCTACAATGACGGC | qPCR |
|  | R: TCTCCTCTATCTGCTCTTCCAGTC |  |
| *ig-d* | F: GCTATCCACACCTATGTCCTCG | qPCR |
|  | R: TCTCACTACTGTTGTCCCTCCAG |  |
| *nitr* | F: GGTCACGATGAGAGACAGAATCC | qPCR |
|  | R: GCAGTTTCAGTGGTTGCCAG |  |
| *gprc*5c | F: CGTTCCATTAGTGGGCGTTTAC | qPCR |
|  | R: AAGGGTGCGTGTGCTGATTG |  |
| *igv* | F: CTGTCAAATCCTCATCTGTTCGTC | qPCR |
|  | R: AAGTCTTGGTTCCTGTCCCAC |  |
| *mhc* | F: GGCAGATGGTGATTCGTATTACC | qPCR |
|  | R: CCAGACCCAAGATTATTTCCG |  |
| *xcr*1l | F: GCATACAAAGACCGAATGTCCAC | qPCR |
|  | R: TAGTTCAGCCTCTCCCACAACC |  |
| *gimap*7l | F: TTCTGTGGTGTAGTGCGAGC | qPCR |
|  | R: CATTTCAAAGAGCGAAGACCTG |  |
| *hsp*90 | F: CTTCCAGAGGAGGCATTTCC | qPCR |
|  | R: GCTGTCATCAGGATTTACCTTGG |  |
| *gimap*4l | F: CAGTGACAGGGAGAGAGATTGC | qPCR |
|  | R: TTTAGCAGTTGTTCCGAGGG |  |
| *il-*15 | F: CTTGTTTGGAATCGGGTTTG | qPCR |
|  | R: ACAGGTTACAGGTGGACTGGAG |  |
| *tgm*3 | F: GATGGAGATGGGAGGATTCTG | qPCR |
|  | R: AGGGTGAAAGGAAGGAGGAGAC |  |
| *il-*21r | F: CGCAGCATCAAGTTTATTCCC | qPCR |
|  | R: TTGTAGTAGGACTCCACTCGCTCC |  |
| *perforin* | F: AGTGAACAAGAACGAGGGCG | qPCR |
|  | R: TTCCTGGTTGATAATCCTGCC |  |
| *il-1t* | F: GCAATCATCCACTGACACTGAAG | qPCR |
|  | R: CACCAAGTTCTCCAGCAGGTAG |  |
| *c*5 | F: TTTCCTTCCTGTTGTAGCGG | qPCR |
|  | R: GGACACCGTATTGACCTTGG |  |
| *tlr*5 | F: CAGTTTGTGCCACTCGTGATAAG | qPCR |
|  | R: GAAGGCTTGTTTCTCCAGTCG |  |
| *cxcl*10 | F: CGAGTCTTGGATTCAGGTTGG | qPCR |
|  | R: GAACAGTGGTCTTCGCTATTGC |  |
| *aid* | F: TCCTACGCTATTTGGGAGCC | qPCR |
|  | R: GACCAGTCTAATGGAGCAGTTGAC |  |
| β-actin | F: ATGTCGCCCTGGACTTCG | qPCR |
|  | R: CTGGGCAACGGAACCTCT |  |
| *ef*2 | F: GACCACCACCACGATGAATG | qPCR |
|  | R: TGGATGTTACCAAGGGAGTGC |  |
| 16S rDNA | F: TACGGCTACCTTGTTACGACTT | 16S rDNA |
|  | R: AGAGTTTGATCCTGGCTCAG |  |
| Probe | GGAGACTGGAATTCCTGGTGTAGCG | FISH |

**Supplementary Table 2** Transcriptome sequencing statistics

| **Sample** | **Read Number** | **Base Number** | **GC Content** | **%≥Q30** |
| --- | --- | --- | --- | --- |
| D1-Spleen | 24,573,056 | 7,347,823,232 | 47.38% | 94.55% |
| D2-Spleen | 23,882,380 | 7,127,572,862 | 47.51% | 94.52% |
| D3-Spleen | 25,091,265 | 7,486,997,564 | 47.37% | 94.46% |
| H1-Spleen | 20,121,853 | 6,023,317,298 | 46.27% | 93.84% |
| H2-Spleen | 23,455,831 | 7,001,744,068 | 46.71% | 94.12% |
| H3-Spleen | 21,550,278 | 6,450,095,360 | 46.89% | 94.09% |

**Supplementary Table 3** Statistical summary of assembly result

| **Length Range** | **Transcript** | **Unigene** |
| --- | --- | --- |
| 300-500 | 27,514(23.25%) | 22,442(40.65%) |
| 500-1000 | 23,622(19.96%) | 12,778(23.15%) |
| 1000-2000 | 25,777(21.78%) | 8,179(14.82%) |
| 2000+ | 41,427(35.01%) | 11,804(21.38%) |
| Total Number | 118,340 | 55,203 |
| N50 Length | 3,077 | 2,762 |
| Mean Length | 1860.05 | 1357.18 |

**Supplementary Table 4** List of KEGG enrichment pathways for DEGs

| #Pathway | ko_ID | DEG_in_Pathway | P-value | Corrected_P-value |
| --- | --- | --- | --- | --- |
| Cytokine-cytokine receptor interaction | ko04060 | 59 | 3.91E-12 | 6.65E-10 |
| Cell adhesion molecules (CAMs) | ko04514 | 41 | 1.06E-05 | 0.001809 |
| Lysosome | ko04142 | 39 | 0.000127 | 0.02155 |
| Sphingolipid metabolism | ko00600 | 14 | 0.001601 | 0.272241 |
| Phagosome | ko04145 | 42 | 0.001723 | 0.292932 |
| Neuroactive ligand-receptor interaction | ko04080 | 36 | 0.001855 | 0.315392 |
| AGE-RAGE signaling pathway in diabetic complications | ko04933 | 33 | 0.002409 | 0.409577 |
| Retinol metabolism | ko00830 | 13 | 0.003215 | 0.546527 |
| Intestinal immune network for IgA production | ko04672 | 13 | 0.003215 | 0.546527 |
| Primary bile acid biosynthesis | ko00120 | 6 | 0.003647 | 0.619926 |
| ABC transporters | ko02010 | 13 | 0.00472 | 0.802427 |
| Pentose and glucuronate interconversions | ko00040 | 11 | 0.005753 | 0.977986 |
| Nitrogen metabolism | ko00910 | 8 | 0.006682 | 1 |
| Starch and sucrose metabolism | ko00500 | 15 | 0.014401 | 1 |
| Glycine, serine and threonine metabolism | ko00260 | 12 | 0.014818 | 1 |
| Fructose and mannose metabolism | ko00051 | 15 | 0.018551 | 1 |
| Porphyrin and chlorophyll metabolism | ko00860 | 8 | 0.021127 | 1 |
| Pentose phosphate pathway | ko00030 | 13 | 0.022438 | 1 |
| PPAR signaling pathway | ko03320 | 16 | 0.022477 | 1 |
| Focal adhesion | ko04510 | 50 | 0.023261 | 1 |
| Glycerolipid metabolism | ko00561 | 18 | 0.024906 | 1 |
| p53 signaling pathway | ko04115 | 16 | 0.03103 | 1 |
| Glycosaminoglycan degradation | ko00531 | 6 | 0.031808 | 1 |
| Ubiquinone and other terpenoid-quinone biosynthesis | ko00130 | 4 | 0.033366 | 1 |
| Endocytosis | ko04144 | 60 | 0.03819 | 1 |
| Arachidonic acid metabolism | ko00590 | 11 | 0.040065 | 1 |
| Regulation of actin cytoskeleton | ko04810 | 45 | 0.04082 | 1 |
| MAPK signaling pathway | ko04010 | 52 | 0.04833 | 1 |
| Arginine biosynthesis | ko00220 | 8 | 0.049938 | 1 |
| Arginine and proline metabolism | ko00330 | 14 | 0.052569 | 1 |
| NOD-like receptor signaling pathway | ko04621 | 14 | 0.057862 | 1 |
| Biosynthesis of amino acids | ko01230 | 28 | 0.059515 | 1 |
| Glycosphingolipid biosynthesis - ganglio series | ko00604 | 4 | 0.059632 | 1 |
| ECM-receptor interaction | ko04512 | 17 | 0.060439 | 1 |
| Ether lipid metabolism | ko00565 | 9 | 0.063612 | 1 |
| Other glycan degradation | ko00511 | 5 | 0.066935 | 1 |
| Glycolysis / Gluconeogenesis | ko00010 | 23 | 0.072477 | 1 |
| Glycosaminoglycan biosynthesis - keratan sulfate | ko00533 | 5 | 0.080851 | 1 |
| Toll-like receptor signaling pathway | ko04620 | 19 | 0.08122 | 1 |
| Butirosin and neomycin biosynthesis | ko00524 | 3 | 0.082008 | 1 |
| Glycerophospholipid metabolism | ko00564 | 21 | 0.084703 | 1 |
| TGF-beta signaling pathway | ko04350 | 19 | 0.112553 | 1 |
| Phosphatidylinositol signaling system | ko04070 | 24 | 0.113397 | 1 |
| GnRH signaling pathway | ko04912 | 21 | 0.121444 | 1 |
| Mucin type O-Glycan biosynthesis | ko00512 | 6 | 0.123638 | 1 |
| Tight junction | ko04530 | 32 | 0.140333 | 1 |
| Apoptosis | ko04210 | 28 | 0.14375 | 1 |
| Amino sugar and nucleotide sugar metabolism | ko00520 | 14 | 0.149728 | 1 |
| DNA replication | ko03030 | 7 | 0.160294 | 1 |
| Galactose metabolism | ko00052 | 9 | 0.160883 | 1 |
| Steroid biosynthesis | ko00100 | 5 | 0.170223 | 1 |
| Cell cycle | ko04110 | 22 | 0.178052 | 1 |
| Adipocytokine signaling pathway | ko04920 | 15 | 0.18661 | 1 |
| Folate biosynthesis | ko00790 | 3 | 0.196051 | 1 |
| Glycosphingolipid biosynthesis - globo series | ko00603 | 3 | 0.196051 | 1 |
| Salmonella infection | ko05132 | 16 | 0.20219 | 1 |
| VEGF signaling pathway | ko04370 | 14 | 0.203091 | 1 |
| ErbB signaling pathway | ko04012 | 19 | 0.233723 | 1 |
| Selenocompound metabolism | ko00450 | 4 | 0.236214 | 1 |
| Fatty acid biosynthesis | ko00061 | 5 | 0.236438 | 1 |
| RIG-I-like receptor signaling pathway | ko04622 | 12 | 0.241601 | 1 |
| Glutathione metabolism | ko00480 | 12 | 0.241601 | 1 |
| Alanine, aspartate and glutamate metabolism | ko00250 | 10 | 0.242937 | 1 |
| Adherens junction | ko04520 | 20 | 0.2566 | 1 |
| Steroid hormone biosynthesis | ko00140 | 5 | 0.283952 | 1 |
| Cytosolic DNA-sensing pathway | ko04623 | 7 | 0.286623 | 1 |
| Phenylalanine metabolism | ko00360 | 3 | 0.296736 | 1 |
| Circadian rhythm | ko04710 | 1 | 0.317017 | 1 |
| Olfactory transduction | ko04740 | 1 | 0.317017 | 1 |
| Hippo signaling pathway -multiple species | ko04392 | 1 | 0.317017 | 1 |
| Linoleic acid metabolism | ko00591 | 3 | 0.331195 | 1 |
| mTOR signaling pathway | ko04150 | 29 | 0.347472 | 1 |
| Inositol phosphate metabolism | ko00562 | 15 | 0.357419 | 1 |
| Drug metabolism - other enzymes | ko00983 | 6 | 0.363602 | 1 |
| Vascular smooth muscle contraction | ko04270 | 21 | 0.388212 | 1 |
| Aldosterone synthesis and secretion | ko04925 | 1 | 0.398555 | 1 |
| Thiamine metabolism | ko00730 | 1 | 0.398555 | 1 |
| Valine, leucine and isoleucine biosynthesis | ko00290 | 1 | 0.398555 | 1 |
| FoxO signaling pathway | ko04068 | 27 | 0.418837 | 1 |
| Herpes simplex infection | ko05168 | 28 | 0.426438 | 1 |
| Sulfur metabolism | ko00920 | 3 | 0.433234 | 1 |
| D-Glutamine and D-glutamate metabolism | ko00471 | 1 | 0.470369 | 1 |
| Ascorbate and aldarate metabolism | ko00053 | 4 | 0.488589 | 1 |
| Glycosphingolipid biosynthesis - lacto and neolacto series | ko00601 | 3 | 0.498036 | 1 |
| Glycosaminoglycan biosynthesis - chondroitin sulfate / dermatan sulfate | ko00532 | 3 | 0.529003 | 1 |
| One carbon pool by folate | ko00670 | 3 | 0.529003 | 1 |
| Glioma | ko05214 | 1 | 0.533618 | 1 |
| Circadian entrainment | ko04713 | 1 | 0.533618 | 1 |
| Amphetamine addiction | ko05031 | 1 | 0.533618 | 1 |
| Taurine and hypotaurine metabolism | ko00430 | 2 | 0.549436 | 1 |
| Glucagon signaling pathway | ko04922 | 1 | 0.589322 | 1 |
| Tyrosine metabolism | ko00350 | 4 | 0.591136 | 1 |
| Carbon metabolism | ko01200 | 31 | 0.595235 | 1 |
| Other types of O-glycan biosynthesis | ko00514 | 4 | 0.61481 | 1 |
| Adrenergic signaling in cardiomyocytes | ko04261 | 25 | 0.617378 | 1 |
| alpha-Linolenic acid metabolism | ko00592 | 2 | 0.619449 | 1 |
| Calcium signaling pathway | ko04020 | 30 | 0.626525 | 1 |
| SNARE interactions in vesicular transport | ko04130 | 4 | 0.637612 | 1 |
| Neurotrophin signaling pathway | ko04722 | 1 | 0.63838 | 1 |
| Inflammatory mediator regulation of TRP channels | ko04750 | 1 | 0.63838 | 1 |
| Ras signaling pathway | ko04014 | 1 | 0.63838 | 1 |
| Dopaminergic synapse | ko04728 | 1 | 0.63838 | 1 |
| Drug metabolism - cytochrome P450 | ko00982 | 5 | 0.656453 | 1 |
| Insulin signaling pathway | ko04910 | 23 | 0.658272 | 1 |
| Salivary secretion | ko04970 | 1 | 0.681584 | 1 |
| Gastric acid secretion | ko04971 | 1 | 0.681584 | 1 |
| Long-term potentiation | ko04720 | 1 | 0.681584 | 1 |
| Renin secretion | ko04924 | 1 | 0.681584 | 1 |
| Gap junction | ko04540 | 13 | 0.699757 | 1 |
| Regulation of autophagy | ko04140 | 3 | 0.712178 | 1 |
| Terpenoid backbone biosynthesis | ko00900 | 3 | 0.712178 | 1 |
| Rap1 signaling pathway | ko04015 | 1 | 0.719632 | 1 |
| Pertussis | ko05133 | 1 | 0.719632 | 1 |
| beta-Alanine metabolism | ko00410 | 4 | 0.719729 | 1 |
| Nicotinate and nicotinamide metabolism | ko00760 | 3 | 0.75313 | 1 |
| Hippo signaling pathway | ko04390 | 1 | 0.753138 | 1 |
| Phenylalanine, tyrosine and tryptophan biosynthesis | ko00400 | 1 | 0.753138 | 1 |
| Synthesis and degradation of ketone bodies | ko00072 | 1 | 0.753138 | 1 |
| Fatty acid elongation | ko00062 | 3 | 0.77178 | 1 |
| Cysteine and methionine metabolism | ko00270 | 9 | 0.773773 | 1 |
| Histidine metabolism | ko00340 | 2 | 0.779087 | 1 |
| cAMP signaling pathway | ko04024 | 1 | 0.782645 | 1 |
| Estrogen signaling pathway | ko04915 | 1 | 0.782645 | 1 |
| Tuberculosis | ko05152 | 1 | 0.808629 | 1 |
| Non-homologous end-joining | ko03450 | 1 | 0.808629 | 1 |
| Alcoholism | ko05034 | 1 | 0.808629 | 1 |
| Peroxisome | ko04146 | 12 | 0.828796 | 1 |
| Metabolism of xenobiotics by cytochrome P450 | ko00980 | 4 | 0.841093 | 1 |
| Hedgehog signaling pathway | ko04340 | 6 | 0.847507 | 1 |
| Oxytocin signaling pathway | ko04921 | 1 | 0.851657 | 1 |
| cGMP-PKG signaling pathway | ko04022 | 1 | 0.851657 | 1 |
| Dorso-ventral axis formation | ko04320 | 3 | 0.860698 | 1 |
| Butanoate metabolism | ko00650 | 2 | 0.863948 | 1 |
| Mismatch repair | ko03430 | 2 | 0.863948 | 1 |
| Melanogenesis | ko04916 | 13 | 0.874492 | 1 |
| Propanoate metabolism | ko00640 | 3 | 0.882721 | 1 |
| Glyoxylate and dicarboxylate metabolism | ko00630 | 6 | 0.897605 | 1 |
| Tryptophan metabolism | ko00380 | 4 | 0.900114 | 1 |
| Fatty acid metabolism | ko01212 | 6 | 0.910835 | 1 |
| Pyruvate metabolism | ko00620 | 7 | 0.919484 | 1 |
| Pyrimidine metabolism | ko00240 | 11 | 0.924583 | 1 |
| Progesterone-mediated oocyte maturation | ko04914 | 10 | 0.933442 | 1 |
| Phototransduction | ko04744 | 1 | 0.939207 | 1 |
| Cardiac muscle contraction | ko04260 | 8 | 0.947781 | 1 |
| Oocyte meiosis | ko04114 | 12 | 0.952139 | 1 |
| Purine metabolism | ko00230 | 22 | 0.953764 | 1 |
| Base excision repair | ko03410 | 2 | 0.960589 | 1 |
| N-Glycan biosynthesis | ko00510 | 4 | 0.963476 | 1 |
| Biosynthesis of unsaturated fatty acids | ko01040 | 1 | 0.971722 | 1 |
| Wnt signaling pathway | ko04310 | 17 | 0.973033 | 1 |
| Alzheimer's disease | ko05010 | 1 | 0.975111 | 1 |
| 2-Oxocarboxylic acid metabolism | ko01210 | 2 | 0.979371 | 1 |
| Oxidative phosphorylation | ko00190 | 16 | 0.988985 | 1 |
| RNA degradation | ko03018 | 6 | 0.989841 | 1 |
| Basal transcription factors | ko03022 | 1 | 0.992116 | 1 |
| Fatty acid degradation | ko00071 | 4 | 0.992304 | 1 |
| Aminoacyl-tRNA biosynthesis | ko00970 | 3 | 0.992701 | 1 |
| Lysine degradation | ko00310 | 4 | 0.996083 | 1 |
| Notch signaling pathway | ko04330 | 3 | 0.998483 | 1 |
| Nucleotide excision repair | ko03420 | 1 | 0.998983 | 1 |
| Citrate cycle (TCA cycle) | ko00020 | 3 | 0.999286 | 1 |
| Proteasome | ko03050 | 2 | 0.99931 | 1 |
| Ubiquitin mediated proteolysis | ko04120 | 11 | 0.999501 | 1 |
| Fanconi anemia pathway | ko03460 | 1 | 0.999635 | 1 |
| mRNA surveillance pathway | ko03015 | 5 | 0.999853 | 1 |
| Valine, leucine and isoleucine degradation | ko00280 | 1 | 0.999885 | 1 |
| Protein processing in endoplasmic reticulum | ko04141 | 17 | 0.999984 | 1 |
| Ribosome biogenesis in eukaryotes | ko03008 | 2 | 0.999984 | 1 |
| RNA transport | ko03013 | 7 | 1 | 1 |
| Spliceosome | ko03040 | 1 | 1 | 1 |

**Supplementary Table 5** Immune system -related KEGG pathways and DEGs in hybrid snakehead infected with *N. seriolae*.

| **Category/**  **Gene ID** | **Gene name** | **Regulated** | **Description** | **Fold change** |
| --- | --- | --- | --- | --- |
| **Intestinal immune network for IgA production ko04672** | | | | |
| c83427.graph_c0 | *pigr* | up | Polymeric immunoglobulin receptor | 1.47 |
| c83712.graph_c0 | *ccr25* | Up | C-C motif chemokine 25-like | 1.26 |
| c72879.graph_c0 | *aid* | down | single-stranded DNA cytosine deaminase | -2.43 |
| c75028.graph_c1 | *h2-auα* | down | H-2 class II histocompatibility antigen, A-U alpha chain-like | -1.74 |
| c80751.graph_c0 | *baff* | down | tumor necrosis factor ligand superfamily member 13B-like isoform X1 | -1.20 |
| c82897.graph_c0 | *ccr9* | down | C-C chemokine receptor type 9-like | -2.36 |
| c89364.graph_c0 | *pigr like* | down | polymeric immunoglobulin receptor-like | -1.58 |
| c86696.graph_c0 | *mhc ii b* | down | MHC class II antigen beta chain | -2.09 |
| c85281.graph_c2 | *mhc* | down | RLA class II histocompatibility antigen, DP alpha-1 chain-like | -1.26 |
| c89067.graph_c0 | *mhc ii a* | down | mamu class II histocompatibility antigen, DR alpha chain-like | -1.15 |
| c90223.graph_c1 | *h2-esβ* | down | H-2 class II histocompatibility antigen, E-S beta chain-like | -1.39 |
| c89263.graph_c0 | *il-15* | down | interleukin-15 isoform X3 | -1.96 |
| c89257 | *tnfsf13b* | down | tumor necrosis factor receptor superfamily member 13B | -2.04 |
| **NOD-like receptor signaling pathway**  **ko04621** | | | | |
| c80526.graph_c0 | *il-8* | up | interleukin 8 | 4.45 |
| c82102.graph_c0 | *tnfα* | up | tumor necrosis factor-like | 1.90 |
| c82873.graph_c0 | *iκbα* | up | NF-kappa-B inhibitor alpha-like | 1.03 |
| c90754.graph_c0 | *iκb* | up | NF-kappa-B inhibitor alpha | 1.47 |
| c86087.graph_c1 | *tnfip3* | up | tumor necrosis factor alpha-induced protein 3 | 1.63 |
| c89079.graph_c1 | *hsp90* | up | heat shock protein HSP 90-alpha | 2.21 |
| c93388.graph_c0 | *rip2* | up | receptor-interacting serine/threonine-protein kinase 2 isoform X1 | 1.14 |
| c90724.graph_c0 | *il-1β* | up | interleukin-1 beta-like | 5.83 |
| c83810.graph_c0 | *il-8* | down | interleukin 8 | -1.37 |
| c86356.graph_c0 | *nlrp3* | down | NACHT, LRR and PYD domains-containing protein 3-like | -1.07 |
| c59838.graph_c0 | *nlrp1b* | down | NACHT, LRR and PYD domains-containing protein 1b allele 2-like | -2.25 |
| c80305.graph_c0 | *mcp-1* | down | eotaxin-like | -3.36 |
| c85788.graph_c0 | *jnk* | down | mitogen-activated protein kinase 8 isoform X3 | -1.12 |
| c86574.graph_c0 | *p38* | down | Mitogen-activated protein kinase 13 | -1.12 |
| **Toll-like receptor signaling pathway** **ko04620** | | | | |
| c80526.graph_c0 | *il-8* | up | interleukin 8 | 4.45 |
| c82102.graph_c0 | *tnfα* | up | tumor necrosis factor-like | 1.90 |
| c82625.graph_c1 | *ap-1* | up | transcription factor AP-1-like | 2.58 |
| c82873.graph_c0 | *iκbα* | up | NF-kappa-B inhibitor alpha-like | 1.03 |
| c90754.graph_c1 | *iκb* | up | NF-kappa-B inhibitor alpha | 1.47 |
| c85734.graph_c0 | *tlr5* | up | toll-like receptor 5 | 4.69 |
| c90836.graph_c0 | *p13k* | up | phosphatidylinositol 4,5-bisphosphate 3-kinase catalytic subunit beta isoform | 1.88 |
| c90724.graph_c0 | *il-1β* | up | interleukin-1 beta-like | 5.83 |
| c92492.graph_c0 | *tlr5* | up | membrane form of toll-like receptor 5 | 1.64 |
| c75066.graph_c0 | *cxcl9* | down | C-X-C motif chemokine 9-like | -2.48 |
| c76533.graph_c0 | *cxcl10* | down | C-X-C motif chemokine 10-like | -1.50 |
| c82047.graph_c0 | *map2k6* | down | dual specificity mitogen-activated protein kinase kinase 6-like | -1.40 |
| c83810.graph_c0 | *il-8* | down | interleukin 8 | -1.37 |
| c84598.graph_c0 | *il-12* | down | interleukin-12 subunit beta-like | -1.39 |
| c85788.graph_c0 | *jnk* | down | mitogen-activated protein kinase 8 isoform X3 | -1.12 |
| c86574.graph_c0 | *p38* | down | Mitogen-activated protein kinase 13 | -1.12 |
| c91102.graph_c0 | *tlr9* | down | toll-like receptor 9 | -2.63 |
| c91288.graph_c0 | *tirap* | down | toll/interleukin-1 receptor domain-containing adapter protein | -1.63 |
| c93773.graph_c2 | *p13k* | down | phosphatidylinositol 4,5-bisphosphate 3-kinase catalytic subunit delta isoform | -1.27 |
| **RIG-I-like receptor signaling pathway** **ko04622** | | | | |
| c80526.graph_c0 | *il-8* | up | interleukin 8 | 4.45 |
| c88486.graph_c0 | *cyld* | up | ubiquitin carboxyl-terminal hydrolase CYLD-like isoform X1 | 3.40 |
| c82102.graph_c0 | *tnfα* | up | tumor necrosis factor-like | 1.90 |
| c82873.graph_c0 | *iκbα* | up | NF-kappa-B inhibitor alpha-like | 1.03 |
| c90754.graph_c1 | *iκb* | up | NF-kappa-B inhibitor alpha | 1.47 |
| c75066.graph_c0 | *cxcl9* | down | C-X-C motif chemokine 9-like | -2.48 |
| c78532.graph_c0 | *sntbad* | down | TANK-binding kinase 1-binding protein 1 isoform X1 | -3.15 |
| c76533.graph_c0 | *cxcl10* | down | C-X-C motif chemokine 10-like | -1.50 |
| c83810.graph_c0 | *il-8* | down | interleukin 8 | -1.37 |
| c84598.graph_c0 | *il-12* | down | interleukin-12 subunit beta-like | -1.39 |
| c85788.graph_c0 | *jnk* | down | mitogen-activated protein kinase 8 isoform X3 | -1.12 |
| **Cytosolic DNA-sensing pathway** **ko04060** | | | | |
| c82873.graph_c0 | *iκbα* | up | NF-kappa-B inhibitor alpha-like | 1.03 |
| c90754.graph_c1 | *iκb* | up | NF-kappa-B inhibitor alpha | 1.47 |
| c89878.graph_c0 | *rip3* | up | receptor-interacting serine/threonine-protein kinase 3-like isoform X2 | 2.30 |
| c90724.graph_c0 | *il-1β* | up | interleukin-1 beta-like | 5.83 |
| c75066.graph_c0 | *cxcl9* | down | C-X-C motif chemokine 9-like | -2.48 |
| c76533.graph_c0 | *cxcl10* | down | C-X-C motif chemokine 10-like | -1.50 |
| c90186.graph_c1 | *cgas* | down | cyclic GMP-AMP synthase | -1.22 |
| c86574.graph_c0 | *p38* | down | Mitogen-activated protein kinase 13 | -1.12 |
